# AMPK Therapy—A Little Goes A Long Way

**DOI:** 10.3390/cells15161503

**Published:** 2026-08-20

**Authors:** Hannah Ceballos, Eryun Zhang, Wendong Huang

**Affiliations:** 1 Department of Diabetes Complications, Beckman Research Institute of City of Hope, Duarte, CA 91010, USA; 2 Arthur Riggs Diabetes and Metabolism Research Institute, City of Hope, Duarte, CA 91010, USA; 3 Irell & Manella Graduate School of Biomedical Science, City of Hope National Medical Center, Duarte, CA 91010, USA

**Keywords:** AMP-activated protein kinase, AMPK, energy metabolism, metabolic diseases, direct AMPK activators, metformin, tissue-specific signaling

## Abstract

**Highlights:**

**What are the main findings?**

- AMPK activity is regulated not only by cellular energy status but also by upstream kinases, post-translational modifications, heterotrimer composition, and subcellular compartmentalization.
- Recent advances in indirect, direct, isoform-selective, and compartment-selective AMPK activators reveal substantial tissue- and context-dependent differences in therapeutic responses.

**What are the implications of the main findings?**

- Precision modulation of AMPK may improve therapeutic efficacy while minimizing adverse effects associated with sustained systemic activation.
- Tissue-, isoform-, and compartment-selective targeting of AMPK represents a promising direction for therapies for metabolic, cardiovascular, neurodegenerative, inflammatory, and age-associated diseases.

**Abstract:**

AMP-activated protein kinase (AMPK) is a highly conserved serine/threonine kinase that integrates energetic, nutrient, hormonal, redox, and stress signals to coordinate cellular and whole-body energy homeostasis. Although AMPK was initially characterized primarily as a sensor of changes in cellular AMP/ATP ratios, recent studies have revealed additional layers of regulation involving upstream kinases, post-translational modifications, heterotrimeric isoform composition, subcellular compartmentalization, and tissue-specific signaling. In this review, we provide an updated overview of the molecular mechanisms regulating AMPK activity, its major downstream metabolic and homeostatic pathways, its roles in metabolic, cardiovascular, neurodegenerative, muscular, malignant, and age-associated diseases, and current strategies for pharmacological AMPK modulation. We compare indirect activators, including metformin and naturally derived compounds, with direct small-molecule agonists targeting the allosteric drug and metabolite (ADaM) site, and emerging activators that selectively engage specific AMPK isoforms, tissues, or subcellular pools. We also discuss endogenous AMPK regulators, including microbiota-derived metabolites, and critically evaluate the potential adverse consequences of sustained or systemic AMPK activation. Collectively, current evidence indicates that the therapeutic effects of AMPK activation are highly dependent on tissue, heterotrimer composition, subcellular localization, disease stage, and the magnitude and duration of activation. Rather than indiscriminate systemic activation, future AMPK-directed therapies are therefore likely to benefit from isoform-, tissue-, and compartment-selective approaches that preferentially engage disease-relevant AMPK signaling while minimizing off-target effects. Continued characterization of AMPK signaling specificity and the development of selective pharmacological modulators should facilitate the translation of AMPK biology into more precise therapies for metabolic and other chronic diseases.

## 1. Introduction

AMP-activated protein kinase (AMPK) is a highly conserved serine/threonine kinase that is a master regulator of cellular and systemic energy homeostasis. AMPK acts as a metabolic checkpoint that enables cells to sense energetic stress and rapidly adapt by coordinating anabolic and catabolic pathways. Under conditions of nutrient deprivation, hypoxia, oxidative stress, or increased energy demand, AMPK restores metabolic balance by promoting ATP-generating processes while simultaneously suppressing energy-consuming biosynthetic pathways. Through these actions, AMPK maintains cellular viability and metabolic flexibility across diverse situations.

Beyond its role as an energy sensor, AMPK is a signaling hub that integrates metabolic, nutrient, redox, inflammatory, and mechanical cues. Indeed, AMPK activation is no longer viewed solely as a consequence of changes in intracellular AMP or ADP concentrations. Instead, AMPK responds to multiple upstream signals, including lysosomal nutrient sensing, glucose starvation, reactive oxygen species (ROS), mitochondrial dysfunction, DNA damage, hypoxia, circadian rhythms, and mechanical stress. This positions AMPK as an integrated stress sensor that links environmental and intracellular signals to coordinated metabolic responses.

AMPK regulates numerous biological processes extending well beyond energy metabolism. Activation of AMPK stimulates glucose uptake, fatty acid oxidation, mitochondrial biogenesis, autophagy, mitophagy, and antioxidant defense while inhibiting protein, lipid, cholesterol, and glucose production. Through interactions with signaling pathways, including mTOR, SIRT1, FOXO, PGC-1α, ULK1, and NF-κB, AMPK coordinates mitochondrial quality control, inflammatory responses, cellular growth, and stress resistance. Consequently, AMPK plays essential roles in maintaining metabolic homeostasis in the liver, skeletal muscle, adipose tissue, heart, intestine, and central nervous system.

Further, AMPK functions beyond individual cells by coordinating communication between tissues and organ systems. In particular, the intestinal microbiota is an important regulator of AMPK signaling through microbial metabolites such as short-chain fatty acids, bile acids, and tryptophan-derived indoles. Conversely, intestinal AMPK shapes gut microbial composition by regulating epithelial barrier integrity, antimicrobial peptide production, and mucosal immunity. These bidirectional interactions establish a host–microbiome signaling network that influences systemic metabolism through gut–liver, gut–brain, and intestine–brown adipose tissue axes, substantially broadening the biological significance of AMPK.

Expectedly, dysregulation of AMPK signaling contributes to the pathogenesis of numerous diseases, including obesity, T2D, metabolic dysfunction-associated steatotic liver disease (MASLD), cardiovascular disease, neurodegenerative disorders, inflammatory diseases, cancer, and aging. As a result, AMPK is being leveraged in metabolic medicine. Metformin, an indirect AMPK activator, has been found to be safe and useful in certain diseases. Highly selective direct AMPK activators targeting the allosteric drug and metabolite (ADaM) site are in development. At the same time, increasing appreciation of tissue-specific AMPK isoform function and inter-organ communication has shifted the field from systemic activation toward localized therapies.

In this review, we provide an updated overview of the molecular mechanisms governing AMPK activation and regulation, its physiological and pathological functions, and its potential as a therapeutic target. In addition to canonical regulation by cellular adenine nucleotides and upstream kinases, we discuss emerging regulatory mechanisms involving post-translational modifications, redox signaling, and subcellular compartmentalization. We further highlight the functional specialization of distinct AMPK heterotrimeric complexes and how their isoform composition, tissue distribution, and intracellular localization can shape substrate specificity and downstream biological responses. We examine the roles of AMPK signaling in metabolic, cardiovascular, inflammatory, neurodegenerative, malignant, and age-associated diseases and critically evaluate both the therapeutic benefits and potential adverse consequences of AMPK activation. Finally, we discuss recent advances in indirect and direct AMPK activators, with particular emphasis on emerging isoform-, tissue-, and compartment-selective strategies. Together, these advances support a shift from systemic AMPK activation toward precision approaches that selectively target disease-relevant AMPK complexes and signaling pools.

### 1.1. AMPK as a Master Nutrition and Energy Sensor

All cells require a high amount of energy to maintain their structure, move, grow, divide, and adapt to their environment. One key requirement for cells is a sensor to help maintain nutrient and energy homeostasis and to help cells adapt during periods of low nutrients. During periods of low nutrients, the AMPK pathway restores energy balance during metabolic stress by regulating its downstream targets to help restore energy and metabolism homeostasis. The AMPK pathway is involved in cellular and whole-body energy metabolism, as well as energy balance, to regulate other pathways in the body [1,2,3].

**Figure 1:**
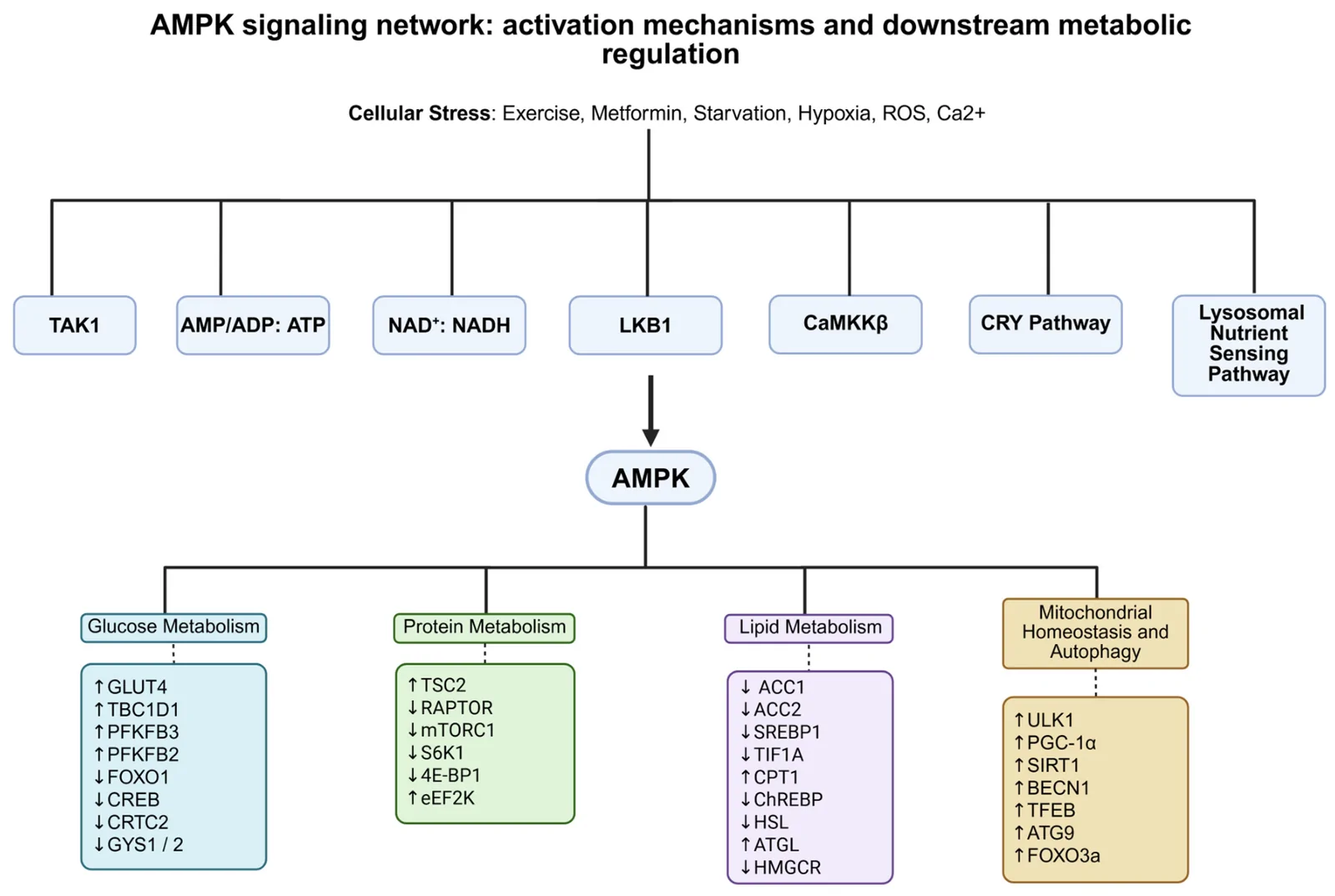
AMPK Signaling Network: Activation Mechanisms and Downstream Metabolic Regulation. Once activated in response to intracellular AMP or ADP concentrations, lysosomal nutrient sensing, glucose starvation, reactive oxygen species, mitochondrial dysfunction, DNA damage, hypoxia, circadian rhythms, or mechanical stress, the AMP-activated Protein Kinase (AMPK) complex phosphorylates key targets to mediate the metabolic effects. The first row of boxes after the activation of AMPK are the pathways (glucose metabolism, protein metabolism, lipid metabolism, and mitochondrial homeostasis and autophagy) that are modulated by AMPK through the activation (↑) or inhibition (↓) of the targeted proteins. Abbreviations: GLUT4, Glucose Transporter Type 4; TBC1D1, TBC1 Domain Family Member 1; TAK1, Transforming Growth Factor-β-Activated Kinase 1; PFKFB3, 6-Phosphofructo-2-Kinase/Fructose-2,6-Biphosphatase 3; PFKFB2, 6-Phosphofructo-2-Kinase/Fructose-2,6-Biphosphatase 2; FOXO1, Forkhead Box Protein O1; CREB, cAMP Response Element-Binding Protein; CRTC2, CREB-Regulated Transcription Coactivator 2; GYS1, Glycogen Synthase 1; GYS2, Glycogen Synthase 2; TSC2, Tuberous Sclerosis Complex 2; RAPTOR, Regulatory-Associated Protein Of mTOR; mTORC1, Mammalian Target Of Rapamycin Complex 1; S6K1, Ribosomal Protein S6 Kinase Beta-1; 4E-BP1, Eukaryotic Translation Initiation Factor 4E-Binding Protein 1; eEF2K, Eukaryotic Elongation Factor 2 Kinase; ACC1, Acetyl-CoA Carboxylase 1; ACC2, Acetyl-CoA Carboxylase 2; SREBP1, Sterol Regulatory Element-Binding Protein 1; TIF1A, Transcription Initiation Factor 1A; CPT1, Carnitine Palmitoyltransferase 1; ChREBP, Carbohydrate Response Element-Binding Protein; HSL, Hormone-Sensitive Lipase; ATGL, Adipose Triglyceride Lipase; HMGCR, 3-Hydroxy-3-Methylglutaryl-CoA Reductase; ULK1, Unc-51 Like Autophagy Activating Kinase 1; PGC-1α, Peroxisome Proliferator-Activated Receptor Gamma Coactivator 1-alpha; SIRT1, Sirtuin 1; BECN1, Beclin-1; TFEB, Transcription Factor EB; ATG9, Autophagy Related 9; FOXO3a, Forkhead Box Protein O3a. Created in BioRender. Ceballos, H. (2026) https://BioRender.com/4952bpg, accessed on 12 August 2026.

As the master energy sensor within the body, AMPK regulates other pathways for lipid metabolism, mitochondrial biogenesis and autophagy, protein metabolism, and glucose metabolism (Figure 1). When activated, the AMPK pathway promotes catabolic processes, such as fatty acid oxidation, glucose uptake, glycolysis, autophagy, mitophagy, and mitochondrial fusion. In the same way, certain anabolic processes are inhibited by the activation of the AMPK pathway, such as protein synthesis, fatty acid synthesis, sterol synthesis, glycogen synthesis and rRNA synthesis. One of the reasons that the AMPK pathway promotes these processes and inhibits the anabolic pathways is to allow ATP levels to be restored during metabolic stress by inhibiting these ATP-consuming pathways and promoting the catabolic pathways that promote ATP generation by breaking down the other macromolecules needed for their pathways [4,5]. This allows the AMPK pathway to be the master energy sensor by being able to convert and divert energy to specific pathways to support ATP levels in the cells.

### 1.2. The Missing Enzyme

The finding that liver acetyl-CoA carboxylase was inactivated in the presence of ATP [6] and that HMG-CoA (3-hydroxy-3-methylglutaryl-CoA) was reduced in the presence of ATP [7] ultimately led to the identification of AMP-activated protein kinase as the responsible enzyme.

### 1.3. Rationale for Therapeutic Targeting

Being a regulator of multiple pathways, AMPK has the potential for treating metabolic diseases, including diabetes, obesity, fatty liver disease, and diseases with metabolic components, such as cancer, cardiovascular disease, and neurodegenerative diseases. The AMPK pathway increases glucose uptake, fatty acid oxidation, mitochondrial biogenesis and mitochondria autophagy while suppressing the synthesis of fatty acids, cholesterol and proteins [1,8]. These facets of AMPK make it a suitable therapeutic target. Some suggest that AMPK may also be targeted for therapeutic effects in immune diseases [9,10].

### 1.4. Literature Search Strategy

This narrative review was informed by a structured literature search of PubMed/MEDLINE, Web of Science, and Google Scholar for studies relevant to AMPK regulation, physiological function, disease pathogenesis, and therapeutic targeting. Searches were performed through 03/2026 using combinations of terms, including “AMPK”, “AMP-activated protein kinase”, “AMPK activation”, “AMPK inhibitor”, “AMPK activator”, “metformin”, “direct AMPK activator”, “ADaM site”, “AMPK isoform”, “AMPK heterotrimer”, “subcellular AMPK”, “lysosomal AMPK”, “AMPK disease”, “AMPK metabolism”, and related disease-specific terms. Priority was given to primary mechanistic studies, clinical studies, and recent comprehensive reviews, particularly the literature published from 2020 onward, while seminal earlier studies were retained where necessary to describe foundational mechanisms. Additional references were identified from the bibliographies of relevant articles. Articles were selected on the basis of relevance to the molecular regulation, physiological functions, disease associations, and therapeutic modulation of AMPK. Because this article is a narrative rather than a formal systematic review or meta-analysis, no quantitative evidence synthesis was performed.

## 2. Molecular Mechanisms of AMPK Activation

### 2.1. Structure of the AMPK Complex

AMPK is a heterotrimer of three subunits α, β, and γ [11,12,13]. The α-subunit α1 and α2 are encoded by *PRKAA1* and *PRKAA2*, the β-subunits β1 and β2 are encoded by *PRKAB1* and *PRKAB2*, and the γ-subunits γ1, γ2, and γ3 are encoded by *PRKAG1*, *PRKAG2*, and *PRKAG3* [14,15] (Table 1). The α-subunit contains the catalytic kinase domain and the critical Thr172 residue, whose phosphorylation is required for full AMPK activation. The β-subunit functions as a scaffold linking the α- and γ-subunits and contains a carbohydrate-binding module that contributes to glycogen binding and forms part of the allosteric drug and metabolite (ADaM) site. The γ-subunit contains cystathionine β-synthase (CBS) domains that bind adenine nucleotides, enabling AMPK to sense changes in cellular AMP, ADP, and ATP concentrations [16].

Combinatorial assembly of these isoforms can generate at least 12 distinct AMPK heterotrimeric complexes, which exhibit differences in biochemical properties, tissue distribution, and physiological functions [17]. Importantly, the heterotrimer composition also contributes to the spatial organization and functional specialization of AMPK signaling, as discussed below.

#### Isoform Composition and Subcellular Compartmentalization of AMPK

AMPK signaling is spatially organized within cells rather than occurring as a uniform cytosolic response. Distinct pools of AMPK have been identified in the cytosol, nucleus, lysosomes, mitochondria, and other membrane-associated compartments, where they can respond to different upstream signals and regulate spatially restricted substrates [3,18,19]. This compartmentalization provides an additional layer of AMPK regulation by coupling local energetic, nutrient, and stress signals to specific downstream responses. For example, during glucose limitation, lysosome-associated AMPK can be activated through recruitment of the AXIN–LKB1 complex to the lysosomal v-ATPase–Ragulator platform, allowing AMPK to respond to nutrient deprivation before substantial changes in whole-cell adenine nucleotide concentrations occur [3,18,20]. In contrast, cytosolic and mitochondria-associated AMPK pools are progressively engaged as energetic stress increases and regulate pathways involved in lipid metabolism, mitochondrial dynamics, and cellular energy homeostasis [3,18,19]. AMPK signaling is also present in the nucleus, where it can influence transcriptional and metabolic programs involved in stress adaptation and cellular homeostasis. Regulated nucleocytoplasmic trafficking provides an additional mechanism for controlling the spatial distribution and function of AMPK signaling [3,19,21].

Subcellular localization is closely linked to the AMPK heterotrimer composition. The two α-, two β-, and three γ-subunit isoforms generate at least 12 AMPK heterotrimers with distinct biochemical properties, tissue-expression patterns, and regulatory characteristics [17]. Differences in isoform composition can influence nucleotide sensitivity, upstream kinase regulation, substrate accessibility, and responsiveness to pharmacological activators [17]. In particular, α1- and α2-containing complexes can exhibit different subcellular distributions and functional properties, although their localization is dynamic and dependent on cell type and physiological context [17,21]. The β-subunit contributes to spatial and functional organization through its carbohydrate-binding module, membrane-associated regulatory mechanisms, and its contribution to the ADaM site, whereas γ-subunit composition influences nucleotide sensing and contributes to tissue-specific functional specialization [17]. Consequently, the biological outcome of AMPK activation depends not only on the magnitude and duration of kinase activity but also on the heterotrimeric complex involved and the subcellular compartment in which activation occurs.

This spatial and isoform-specific organization has important therapeutic implications. Systemic pan-AMPK activation may simultaneously engage AMPK complexes with beneficial and potentially undesirable functions in different tissues and cellular compartments, thereby limiting therapeutic precision. In contrast, targeting specific heterotrimeric complexes or subcellular AMPK pools may preferentially engage disease-relevant signaling pathways while reducing unwanted effects. Compartment-selective activation of lysosomal AMPK provides an emerging example of this strategy, whereas isoform-selective activators demonstrate the feasibility of pharmacologically discriminating among AMPK heterotrimers. Therefore, a more complete understanding of the spatial organization, substrate specificity, and disease-dependent functions of individual AMPK complexes will be important for developing next-generation precision AMPK therapeutics [3,17,22].

### 2.2. Canonical Activation of AMPK

Physiological conditions such as nutrient deprivation, prolonged exercise, ischemia, hypoxia, and mitochondrial dysfunction reduce ATP production while increasing intracellular AMP and ADP concentrations, thereby activating AMPK to restore metabolic balance [1,6,8]. Once activated, AMPK promotes ATP-generating catabolic pathways, including glucose uptake, fatty acid oxidation, and autophagy, while simultaneously suppressing ATP-consuming anabolic processes, such as protein synthesis, lipogenesis, and glycogen synthesis (Table 2).

#### 2.2.1. AMP/ADP-Dependent Nucleotide Sensing

The classical mechanism of AMPK activation depends on changes in intracellular adenine nucleotide concentrations. The AMPK γ-subunit contains four cystathionine β-synthase (CBS) domains that form nucleotide-binding sites capable of sensing fluctuations in AMP, ADP, and ATP concentrations [8,23].

During energetic stress, AMP and ADP compete with ATP for binding to the γ-subunit. AMP activates AMPK through three complementary mechanisms: (i) promoting phosphorylation of Thr172 within the activation loop of the α-subunit by upstream kinases; (ii) protecting Thr172 from protein phosphatase-mediated dephosphorylation; and (iii) directly allosterically increasing kinase activity following phosphorylation 1, 8]. ADP similarly protects Thr172 from dephosphorylation but lacks significant allosteric activation. These mechanisms enable AMPK to respond rapidly to relatively small changes in cellular energy status before severe ATP depletion occurs.

#### 2.2.2. LKB1-Dependent Activation

Phosphorylation of Thr172 by liver kinase B1 (LKB1, also known as STK11) represents the principal mechanism responsible for AMPK activation in most mammalian tissues [1,24]. LKB1 forms a constitutively active heterotrimeric complex with the pseudokinase STRAD and the scaffolding protein MO25 [25]. Assembly of this complex promotes LKB1 stability, cytoplasmic localization, and catalytic activity, enabling efficient phosphorylation of AMPK during stress.

Nucleotide sensing and LKB1-mediated phosphorylation function cooperatively rather than independently. Binding of AMP or ADP markedly enhances the susceptibility of Thr172 to LKB1 phosphorylation while protecting it from dephosphorylation [1,8]. In addition to regulating metabolism, LKB1 is a tumor suppressor whose inactivation contributes to metabolic dysregulation and tumorigenesis, highlighting the relationship between energy sensing and cancer formation [24,26].

#### 2.2.3. CaMKKβ-Mediated Activation

AMPK can also be activated through calcium-dependent signaling mediated by calcium/calmodulin-dependent protein kinase β (CaMKKβ, also known as CaMKK2). Unlike LKB1, CaMKKβ phosphorylates Thr172 independently of changes in intracellular AMP or ADP concentrations, thereby coupling calcium signaling directly to metabolic regulation [27,28].

Transient increases in intracellular Ca^2+^ induced by G protein-coupled receptors, receptor tyrosine kinases, neuronal activity, muscle contraction, and immune cell activation stimulate CaMKKβ-dependent AMPK activation. This is particularly important in skeletal muscle, neurons, endothelial cells, and macrophages, where calcium signaling rapidly coordinates metabolic adaptation [27]. Reciprocal regulation between AMPK and calcium homeostasis through phosphorylation of stromal interaction molecule 1 links AMPK activation to regulation of store-operated calcium entry and endoplasmic reticulum calcium signaling [29].

#### 2.2.4. TAK1-Mediated Activation

Transforming growth factor-β-activated kinase 1 (TAK1; MAP3K7) has also been implicated as an upstream regulator of AMPK activation. TAK1 is a member of the mitogen-activated protein kinase kinase kinase (MAP3K) family that integrates signals from inflammatory cytokines and cellular stress. Early studies demonstrated that mammalian TAK1 can phosphorylate AMPK in vitro, and subsequent evidence showed that the activated TAK1–TAB1 complex can directly phosphorylate Thr172 within the activation loop of the AMPK α-subunit, thereby increasing AMPK catalytic activity [30,31,32]. Genetic studies further demonstrated that loss of TAK1 reduces AMPK Thr172 phosphorylation and AMPK activity under conditions of metabolic stress, supporting a functional link between TAK1 and AMPK signaling [32]. However, unlike LKB1 and CaMKK2, which are well-established physiological AMPK, the contribution of TAK1 to AMPK activation appears to be dependent on cell type and stimulus, and whether TAK1 functions broadly as a direct physiological AMPK kinase remains under investigation [31]. Thus, TAK1 may provide an additional context-dependent mechanism connecting inflammatory and stress signaling to AMPK-mediated metabolic adaptation.

#### 2.2.5. Inhibitory Phosphorylation of the AMPK α-Subunit

In addition to activating phosphorylation at Thr172, AMPK activity is negatively regulated by phosphorylation within the serine/threonine-rich (ST) loop of the catalytic α-subunit. Ser485 in AMPKα1 and the corresponding Ser491 residue in AMPKα2 have been identified as regulatory phosphorylation sites [33,34]. Phosphorylation at these sites can antagonize AMPK activation by limiting subsequent phosphorylation of Thr172, thereby providing an additional mechanism for controlling AMPK activity. Insulin and growth-factor signaling through protein kinase B (Akt) can promote inhibitory phosphorylation, particularly at Ser485 of AMPKα1, thereby reducing LKB1-mediated Thr172 phosphorylation and AMPK activation [34]. In addition, cAMP-dependent protein kinase A (PKA) signaling can promote phosphorylation of Ser485/491 and suppress AMPK activity, providing a mechanism for crosstalk between cAMP signaling and cellular energy sensing [35]. Thus, AMPK activity is determined not only by activating phosphorylation at Thr172 through upstream kinases but also by inhibitory phosphorylation of the α-subunit, allowing integration of cellular energy status with hormonal and growth-factor signaling.

Collectively, canonical AMPK regulation involves a balance between activating and inhibitory phosphorylation. Thr172 phosphorylation by upstream kinases, principally LKB1 and CaMKK2, promotes AMPK activation, while TAK1 may provide an additional context-dependent route linking cellular stress and inflammatory signaling to AMPK. Conversely, phosphorylation of Ser485/491 within the AMPK α-subunit through signaling pathways involving Akt and PKA can antagonize Thr172 phosphorylation and suppress AMPK activity. Together, these regulatory mechanisms enable AMPK to integrate cellular energy status with hormonal, growth-factor, calcium, and stress signals.

### 2.3. Beyond AMP/ATP: Emerging Mechanisms Regulating AMPK Activation

Rather than functioning solely as a sensor of cellular energy charge, AMPK is now recognized as an integrated cellular stress sensor that responds to nutrient availability, redox status, organelle function, mechanical forces, and environmental stress. These non-canonical mechanisms enable AMPK to coordinate metabolic adaptation before substantial ATP depletion occurs and considerably broaden its physiological functions [1,6].

#### 2.3.1. Post-Translational Regulation of AMPK

Beyond changes in adenine nucleotide levels and Ca^2+^-dependent signaling, AMPK activity is dynamically regulated by multiple post-translational modifications (PTMs), including phosphorylation, ubiquitination, SUMOylation, acetylation, and redox-dependent modifications [36,37]. These modifications can influence AMPK catalytic activity, protein stability, subcellular localization, and interactions with upstream regulators, providing additional mechanisms through which nutrient, hormonal, and stress signals modulate AMPK function [36,37]. As discussed above, inhibitory phosphorylation of Ser485 in AMPKα1 and the corresponding Ser491 residue in AMPKα2 can antagonize activating Thr172 phosphorylation, thereby linking insulin/Akt and cAMP/PKA signaling to suppression of AMPK activity [33,34,35].

Ubiquitination represents another important regulatory mechanism. Ubiquitination of AMPKα can interfere with its interaction with LKB1 and consequently reduce LKB1-mediated phosphorylation and AMPK activation. Conversely, the deubiquitinase USP10 removes inhibitory ubiquitination from AMPKα, thereby facilitating its phosphorylation by LKB1. During energy stress, AMPK phosphorylates USP10 at Ser76 and enhances USP10 activity, establishing a positive feed-forward loop that amplifies AMPK activation [38]. Ubiquitination and SUMOylation of AMPK subunits can further modulate AMPK stability and activity, highlighting the importance of ubiquitin-related modifications in fine-tuning AMPK signaling [36,37].

Acetylation provides an additional layer of AMPK regulation and may influence AMPK signaling either directly through modification of AMPK subunits or indirectly through its upstream regulatory machinery [1,36,37]. AMPK is also sensitive to oxidative and other redox-dependent modifications. Reactive oxygen species can induce oxidative modification of AMPK, including S-glutathionylation of AMPKα and AMPKβ subunits, thereby altering kinase activity independently of substantial changes in cellular ATP levels [37,39]. Indeed, exposure to H_2_O_2_ has been shown to induce S-glutathionylation and activation of AMPK, illustrating a direct mechanism by which oxidative signals can regulate AMPK independently of classical energy sensing [39]. Importantly, redox regulation of AMPK is context-dependent, and the consequences may vary according to the magnitude and duration of oxidative stress [36,37].

Collectively, these PTMs establish AMPK as a regulatory hub whose activity reflects not only cellular energy charge but also hormonal, proteostatic, and redox signals. Dysregulation of these modifications under pathological conditions may contribute to impaired AMPK signaling and disease progression. Therefore, enzymes controlling AMPK phosphorylation, ubiquitination, deubiquitination, acetylation, SUMOylation, and redox state may provide alternative therapeutic targets for restoring appropriate AMPK activity [36,37].

#### 2.3.2. Redox-Dependent Regulation

Beyond direct oxidative modification of AMPK, changes in cellular redox homeostasis regulate AMPK through several upstream signaling mechanisms. The intracellular NAD^+^/NADH ratio reflects mitochondrial metabolic status and influences AMPK activation through the NAD^+^-dependent deacetylase SIRT1. Increased NAD^+^ availability activates SIRT1, which deacetylates LKB1 and promotes its cytoplasmic localization, thereby enhancing LKB1-dependent phosphorylation of AMPK [40]. Mitochondrial dysfunction and moderate reactive oxygen species (ROS) production activate AMPK through oxidative signaling pathways that promote mitochondrial quality control, antioxidant responses, and mitophagy [41,42].

#### 2.3.3. Lysosomal Nutrient Sensing

A significant advance in AMPK biology has been the discovery that lysosomes function as metabolic signaling platforms. Under glucose starvation, reduced intracellular fructose-1,6-bisphosphate disrupts the interaction between aldolase and the vacuolar ATPase, resulting in recruitment of the Axin–LKB1 complex to the Ragulator scaffold on lysosomal membranes [36,43]. This signaling pathway enables AMPK activation before detectable reductions in cellular ATP, allowing rapid metabolic adaptation to nutrient limitation. These findings demonstrate that glucose itself can be sensed independently of adenine nucleotide levels.

#### 2.3.4. Cellular Stress Signaling

AMPK also integrates multiple environmental stress signals beyond nutrient deprivation. DNA damage activates AMPK through ATM-dependent signaling, coupling genome surveillance with metabolic adaptation and cell-cycle regulation [20]. Hypoxia stimulates AMPK through both ATP-dependent and ROS-mediated mechanisms, reducing anabolic metabolism while promoting cellular survival [44]. Mechanical stress and extracellular matrix stiffness activate AMPK in skeletal muscle, cardiovascular tissues, and cancer cells, where it coordinates cytoskeletal remodeling, mitochondrial metabolism, and mechanotransduction [45]. Osmotic stress likewise activates AMPK, enabling cells to maintain metabolic homeostasis during changes in osmolarity [46].

#### 2.3.5. Circadian Regulation

AMPK phosphorylates circadian clock proteins, including cryptochrome (CRY), regulating clock protein stability and synchronizing cellular metabolism with daily feeding–fasting cycles [47]. Conversely, circadian oscillations in nutrient availability and metabolic activity influence AMPK signaling, establishing a bidirectional communication between energy sensing and circadian regulation.

Collectively, these discoveries have fundamentally reshaped the understanding of AMPK activation. AMPK is now recognized as a master integrator of energetic, nutrient, redox, inflammatory, and mechanical stress signals. This expanded regulatory framework explains the remarkably broad physiological functions of AMPK across multiple tissues and provides a conceptual foundation for the development of next-generation therapeutic strategies targeting metabolic disease, cardiovascular disorders, cancer, neurodegeneration, and aging.

### 2.4. Key Downstream Signaling Pathways

The AMPK pathway activates downstream signaling pathways that are necessary for cellular metabolism. One signaling pathway that is activated is the UNC-51-like kinase 1 (ULK1), which is necessary for mitophagy [48,49]. Mitophagy specifically targets mitochondria in a damaged or defective state, engulfing them by autophagosomes, degrading them and recycling their contents for reuse in the cell. AMPK phosphorylates and activates ULK1, which activates the autophagy pathway; this is achieved by AMPK and ULK1 forming a stable complex that activates the autophagy cascade within the mitochondria. This phosphorylation is necessary for the clearance of dysfunctional mitochondria, which are a source of oxidative stress in the cell (Table 3).

Another important downstream target that AMPK activates is peroxisome proliferator-activated receptor-γ coactivator 1α (PGC-1α). PGC-1α is a coactivator that enhances the activity of transcription factors that are on nuclear-encoded mitochondrial genes. AMPK can directly phosphorylate PGC-1α, which creates a positive feedback loop by causing activation of AMPK, which then phosphorylates PGC-1α.

AMPK pathways also regulate anabolic pathways. Most of these are inhibited through AMPK activation to conserve ATP and ATP production and include lipid, carbohydrate, and protein metabolism. The mTOR signaling pathway, specifically mTOR complex 1 (mTORC1), is involved in protein synthesis [2,50]. AMPK phosphorylates RAPTOR and TSC2, which then activate mTOR for protein synthesis [51]. mTOR signaling is modulated by the phosphorylation of AMPK, and although this phosphorylation does not completely inhibit these enzymes and proteins, it limits the extent of phosphorylation.

## 3. AMPK in Cellular and Metabolic Regulation

### 3.1. Glucose and Lipid Metabolism

To phosphorylate AMPK, there must be a response to stimuli that increases nutrition and energy stress. Stimuli such as glucose and lipids are major sources of energy. To increase ATP levels, AMPK promotes the breakdown and inhibition of glucose and lipid synthesis and storage. AMPK promotes glucose uptake by phosphorylating TBC domain family member 1 (TBC1D1), which controls the translocation of the glucose transporters GLUT4 and GLUT1. Glucose metabolism is partly regulated by AMPK through deregulation of glycolysis. AMPK inhibits storage of glucose in some tissues by inhibiting multiple isoforms of glycogen synthase [52]. It also inhibits the transcriptional induction of glycogenesis through the synthesis of glucose [2].

AMPK directly phosphorylates acetyl-CoA carboxylase 1 (ACC1), which suppresses fatty acid synthesis while promoting fatty acid oxidation. Fatty acid oxidation is also carried out at the mitochondrial outer membrane by acetyl-CoA carboxylase 2 (ACC2). This links the AMPK pathway with lipid metabolism and mitochondrial function. The activation of AMPK inhibits lipogenic transcriptional programs—e.g., sterile regulatory element-binding protein 1 (SREBP1), a transcriptional regulator of lipid synthesis. This limits lipid synthesis and favors oxidation of fatty acids as an energy source. Overall, AMPK activation favors glucose uptake to help promote ATP regeneration and promotes lipid synthesis to improve metabolic function in the cell.

### 3.2. Autophagy and Mitochondrial Function

AMPK helps to regulate mitochondria by controlling the mitochondrial number through stimulation of mitochondrial biogenesis, regulates mitochondrial networks, and regulates mitochondrial function through mitophagy and autophagy. AMPK phosphorylates multiple transcription factors for mitochondrial fission and mitochondrial biogenesis. For mitochondrial biogenesis to occur, there needs to be an upregulation of mitochondrial proteins and an increase in lipid production for new growth and division from the pre-existing mitochondria. For this to occur, transcription factors need to be relayed to the nucleus to increase mitochondrial protein gene expression [6,51].

AMPK also participates in mitochondrial autophagy. Autophagy replenishes nutrient stores during times of starvation and recycles proteins, macromolecules, and organelles. Many of the downstream signaling pathways that AMPK activates by phosphorylating transcription factors are related to mitochondrial autophagy and mitophagy [53]. For example, the ULK1 pathway is phosphorylated directly by AMPK to regulate mitochondrial fission and to drive degradation of damaged mitochondria [27]. After that, it activates the mitochondrial biogenesis pathway to create new mitochondria, thereby stimulating ATP production.

### 3.3. Crosstalk with Inflammation and Stress Signaling

Inflammation is a characteristic of many metabolic diseases, including obesity, NAFLD, cardiovascular disease, insulin resistance, and T2D. Inflammation involves the mobilization of macrophages, neutrophils, mast cells, and T cells along with other immune cells. In the presence of inflammation, AMPK activity is reduced. The reduction in AMPK activity is mostly due to an increase in protein phosphorylation, which leads to the dephosphorylation of Thr172, reducing the activity of AMPK. AMPK phosphorylates transcriptional factors that inhibit the inflammatory responses [24]. When AMPK activity is reduced, inflammatory responses linked to oxidative metabolism and fatty acid synthesis are more active. AMPK reduction also seems to impinge upon macrophages [54].

### 3.4. AMPK and Host–Microbiome Interactions

The intestinal microbiota has emerged as a critical regulator of host metabolism, immunity, and tissue homeostasis, with extensive bidirectional crosstalk between microbial communities and AMPK signaling. AMPK integrates microbial-derived metabolic signals to coordinate epithelial barrier integrity, immune homeostasis, and systemic metabolic adaptation. Conversely, intestinal AMPK influences microbial composition by regulating epithelial metabolism, antimicrobial peptide production, mucus secretion, and barrier function in a dynamic host–microbiome feedback loop [1,6,8].

Microbial metabolites are upstream regulators of AMPK activation. Short-chain fatty acids (SCFAs), particularly butyrate and propionate, produced during bacterial fermentation of dietary fiber, activate AMPK in intestinal epithelial cells through mechanisms involving increased AMP/ATP ratios, G protein-coupled receptor signaling, and histone deacetylase inhibition [55,56]. AMPK activation promotes mitochondrial oxidative metabolism, stimulates autophagy, and enhances epithelial barrier integrity through increased expression and assembly of tight junction proteins, including zonula occludens-1 (ZO-1), occludin, and claudins [57]. In addition, AMPK suppresses NF-κB-mediated inflammatory signaling and promotes anti-inflammatory immune responses, thereby limiting chronic intestinal inflammation and maintaining mucosal immune tolerance [1,6]. Gut microbiota-derived indole metabolites generated from dietary tryptophan activate AMPK directly or indirectly through aryl hydrocarbon receptor (AhR)-dependent pathways, promoting epithelial regeneration, mucosal repair, and intestinal homeostasis [58].

Bile acids constitute another important interface linking the gut microbiota with AMPK signaling. Primary bile acids synthesized in the liver undergo extensive microbial transformation into secondary bile acids that regulate host metabolism through receptors, including farnesoid X receptor (FXR) and Takeda G protein-coupled receptor 5 (TGR5) [59]. Bile acid composition influences AMPK activation in multiple tissues, whereas AMPK itself regulates bile acid synthesis and enterohepatic metabolism, establishing a reciprocal regulatory network that integrates nutrient sensing, microbial metabolism, and host energy homeostasis [1,59]. Dysregulation of this bile acid–microbiota–AMPK axis has been implicated in obesity, metabolic dysfunction-associated steatotic liver disease (MASLD), inflammatory bowel disease (IBD), and colorectal cancer [59].

An important consideration is whether microbiota-dependent AMPK regulation occurs predominantly locally within the intestine or systemically in distal organs. The intestinal epithelium represents a major initial site of exposure because microbial metabolites are generated at high concentrations within the intestinal lumen. Local AMPK activation can regulate epithelial mitochondrial metabolism, autophagy, tight-junction organization, antimicrobial defense, and inflammatory responses. However, microbiota-derived metabolites that enter the portal and systemic circulation can also influence the liver, adipose tissue, skeletal muscle, immune system, and central nervous system. SCFAs, secondary bile acids, and tryptophan metabolites therefore provide potential molecular mediators of gut–liver, gut–adipose, gut–muscle, and gut–brain communication. The relative contribution of local versus systemic AMPK activation is metabolite-, concentration-, tissue-, and context-dependent and remains an important area for further investigation.

The physiological importance of intestinal AMPK is supported by genetic studies demonstrating that disruption of intestinal AMPKα1 alters antimicrobial peptide expression, microbial composition, and microbial metabolites and impairs communication between the intestine and brown adipose tissue. Intestinal AMPK deficiency also reduces the metabolic effects of metformin, supporting a role for the intestinal microbiota–AMPK axis in systemic energy expenditure and glucose homeostasis [60]. Thus, AMPK can function not only as a cellular energy sensor but also as an important component of inter-organ communication linking microbial metabolism with whole-body metabolic regulation. These findings raise the possibility of therapeutically targeting the gut microbiota–AMPK axis. Dietary interventions rich in fermentable fiber can increase microbial SCFA production, whereas dietary phytochemicals and herbal components may reshape microbial communities and their metabolite profiles. As highlighted by Liu et al. [61], dietary herbs may influence obesity-associated metabolic phenotypes through coordinated changes in gut microbial composition and microbial metabolites, providing a potential nutritional approach for manipulating microbiota-dependent metabolic signaling. Probiotics and prebiotics may similarly enrich metabolite-producing microbial communities, whereas postbiotics, including defined SCFAs or other microbial metabolites, could provide more controllable exposure without requiring permanent microbial engraftment. Fecal microbiota transplantation (FMT) represents a broader strategy for restructuring microbial ecosystems, but its effects on AMPK are likely indirect and highly dependent on donor composition, recipient microbiota, diet, and disease state. Consequently, evidence supporting microbiota-directed interventions specifically through AMPK remains substantially stronger in experimental models than in clinical studies.

**Figure 2:**
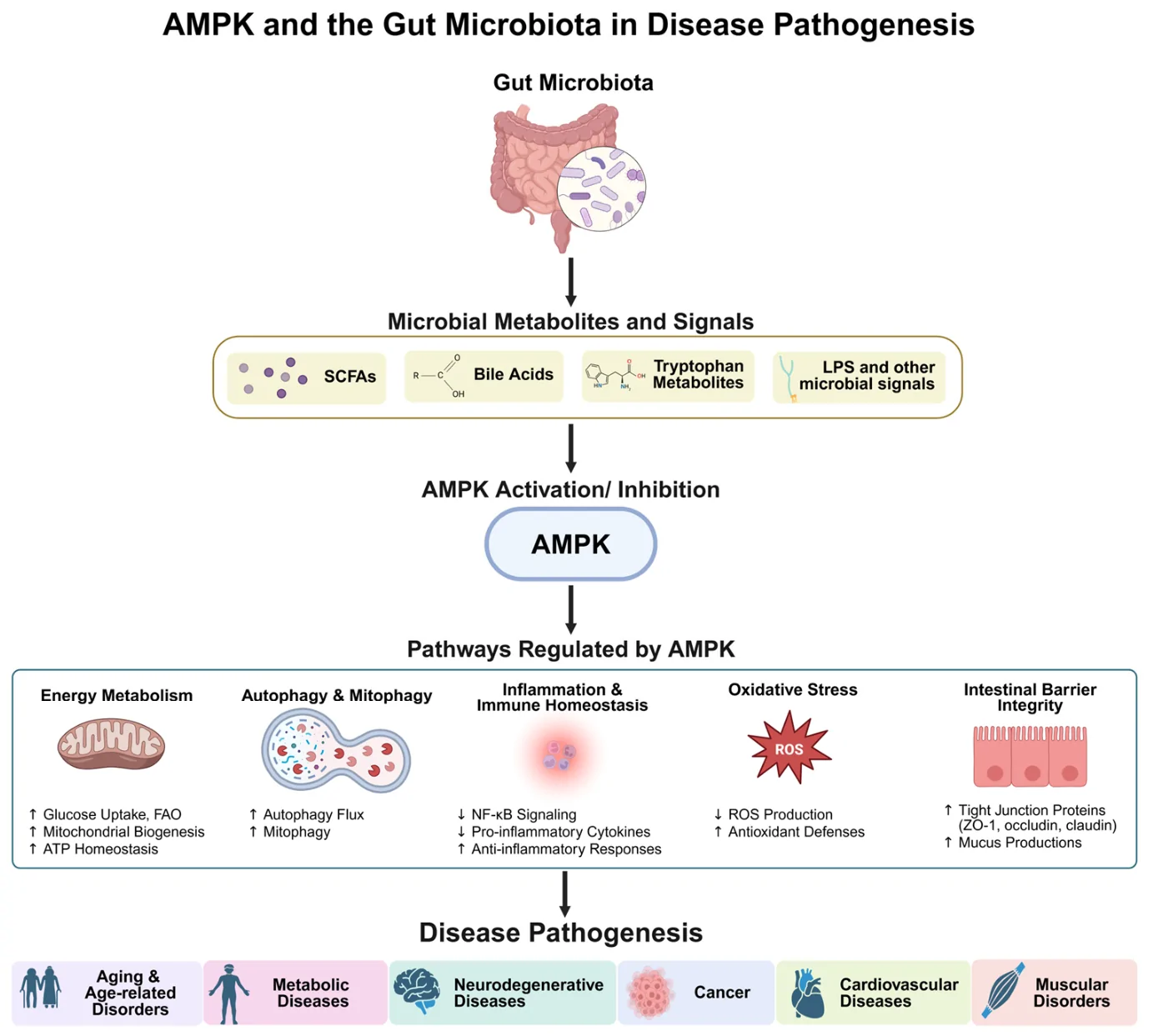
AMPK and the Gut Microbiota in Disease Pathogenesis. Microbiota-dependent AMPK signaling contributes to gut–organ axes, while microbial metabolites are among the major upstream regulators of AMPK activation. AMPK is a master energy sensor and is needed to maintain energy homeostasis. Dysregulation of AMPK signaling drives a range of pathologies, such as cardiovascular disease, cancer, and neurodegenerative diseases. Black downward arrows indicate the direction of signaling and regulatory relationships from the gut microbiota and microbial metabolites to AMPK modulation, downstream AMPK-regulated pathways, and disease pathogenesis. Upward and downward arrows (↑ and ↓) indicate increases and decreases, respectively, in the indicated biological processes. Abbreviations: LPS, lipopolysaccharide; SCFA, short-chain fatty acids. Created in BioRender. Ceballos, H. (2026) ks5deal https://BioRender.com.

Collectively, the gut microbiota–AMPK axis represents a multilayered signaling network in which microbial metabolites can influence AMPK locally in the intestinal epithelium and, following absorption, potentially in distal tissues. SCFAs, secondary bile acids, and tryptophan-derived metabolites illustrate mechanistically distinct routes connecting microbial metabolism with host energy sensing (Figure 2). However, the pleiotropic actions of these metabolites, differences in tissue exposure, and substantial interindividual variability in microbiome composition complicate attribution of therapeutic effects specifically to AMPK. Future studies combining defined microbial communities, metabolomics, tissue-specific AMPK models, and controlled dietary or postbiotic interventions will be important for determining whether this axis can be translated into precise AMPK-directed therapies.

## 4. AMPK in Disease Pathogenesis

### 4.1. Metabolic Diseases

An imbalance in metabolism can lead to the development of diseases such as obesity, T2D, and MASLD. These diseases can arise secondary to dysregulation of mitochondria, lower metabolic function, and inflammation. Many of these diseases are in many ways mediated by AMPK (Figure 2). It is possible that AMPK activators could help to limit or reverse some or all of these conditions.

#### 4.1.1. Type 2 Diabetes

T2D is characterized by elevated blood glucose levels and insulin resistance in the body. T2D leads to and increases the rate of cardiovascular disease, obesity, cancer, and kidney failure. Dysregulation of AMPK may contribute to insulin resistance [62]. In animals, activation of AMPK limits insulin resistance [45]. AMPK activity is reduced in the skeletal muscle of insulin-resistant mice and humans [63,64]. AMPK activators that are being used to treat T2D to improve glucose uptake in the body and lower overall blood glucose levels [65]. Of relevance, AMPK activation increases glucose uptake by muscle [66].

#### 4.1.2. Obesity

Obesity develops under increased energy supply in the body. Under this scenario, adipose tissue stores extra energy [67]. Adipocytes activate AMPK, affecting cholesterol synthesis by promoting lipolysis in the adipocytes and decreasing lipogenesis. Macrophages infiltrate adipose tissue and contribute to insulin resistance [68,69,70]. Interestingly, AMPK can be activated in the adipose tissue to promote mitochondrial fatty acid oxidation and increase energy expenditure [8].

#### 4.1.3. MASLD

Metabolic dysfunction-associated steatotic liver disease (MASLD) is characterized by excessive hepatic lipid accumulation, insulin resistance, inflammation, and progressive liver injury. AMPK is a central regulator of hepatic energy metabolism and regulates lipid homeostasis in part through phosphorylation of acetyl-CoA carboxylase (ACC) [71]. However, liver-specific genetic studies have demonstrated that loss of hepatic AMPK alone is not sufficient to increase de novo lipogenesis or to induce spontaneous hepatic steatosis under basal physiological conditions, indicating that AMPK deficiency is not, by itself, a primary driver of hepatic lipid accumulation [72]. Instead, impaired AMPK signaling may exacerbate hepatic metabolic dysfunction in the presence of nutritional excess, obesity, or insulin resistance, whereas hepatic AMPK activation can protect against diet-induced steatosis [72,73,74]. Conversely, pharmacological or genetic activation of AMPK suppresses hepatic lipogenesis through regulation of ACC and sterol regulatory element-binding protein-1c (SREBP-1c), promotes lipid catabolism, and protects against diet-induced hepatic steatosis and metabolic dysfunction in experimental models [71,72,74,75,76]. These findings suggest that AMPK functions primarily as an adaptive metabolic stress-response pathway that limits the progression of MASLD rather than serving as the sole determinant of hepatic lipid accumulation.

### 4.2. Cardiovascular Disease

When AMPK is activated, glucose uptake, fatty acid oxidation, and glycolysis are properly regulated, but when AMPK is dysregulated, insulin resistance, improper glucose uptake, and larger fatty acid deposits occur not only in the liver but also elsewhere [77]. High blood cholesterol affects cardiac myocytes negatively [78]. Proper AMPK signaling inhibits immune cells and lowers inflammation related to cardiovascular disease [53]. In fact, AMPK activators lessen cardiovascular disease manifestations and alter clinical outcomes [79].

### 4.3. Cancer

AMPK has complex and context-dependent roles in cancer. Traditionally, AMPK has been considered a tumor-suppressive pathway because activation of the LKB1–AMPK axis restrains energy-consuming anabolic processes and inhibits mTORC1-dependent protein synthesis and cell growth [80,81,82]. Consistent with this concept, loss of AMPK activity can enhance aerobic glycolysis, promote the Warburg phenotype, and facilitate tumor growth in certain experimental models. For example, genetic deletion of the AMPKα1 catalytic subunit accelerates Myc-induced lymphomagenesis and promotes a metabolic shift toward aerobic glycolysis, supporting a tumor-suppressive role for AMPK in this context [83]. However, accumulating evidence indicates that AMPK can also support the survival and metabolic adaptation of established tumors under conditions of energetic stress [80,81,82]. Tumor cells frequently encounter nutrient deprivation, hypoxia, oxidative stress, and matrix detachment, conditions under which AMPK can promote metabolic adaptation and maintain cellular energy and redox homeostasis. In particular, AMPK-mediated inhibition of ACC1 and ACC2 reduces NADPH consumption by fatty acid synthesis while promoting fatty acid oxidation, thereby maintaining NADPH levels, limiting oxidative stress, and supporting tumor-cell survival during energy stress [84]. Thus, AMPK should not be viewed exclusively as a tumor suppressor; rather, its effects depend on tumor type, genetic background, disease stage, and metabolic context. These dual functions present an important challenge for AMPK-directed cancer therapy, as AMPK activation may suppress tumor initiation and anabolic growth in some settings while promoting metabolic adaptation and survival of established tumors in others [80,81,82].

### 4.4. Neurodegeneration

AMPK contributes to central regulation of energy homeostasis through its actions in the hypothalamus, where it integrates hormonal and nutrient signals to regulate appetite and whole-body metabolism [85]. Beyond its metabolic functions, AMPK has emerged as an important regulator of neurodegenerative diseases. Aging, hypertension, obesity, diabetes, and metabolic syndrome are major risk factors for neurodegeneration, particularly Alzheimer’s disease (AD), and are frequently associated with impaired energy metabolism, oxidative stress, and mitochondrial dysfunction [36]. AMPK maintains neuronal energy homeostasis by promoting mitochondrial biogenesis, mitochondrial quality control, autophagy, and mitophagy through downstream effectors, including PGC-1α, ULK1, and TFEB [1]. In experimental models of AD, AMPK activation has been shown to improve mitochondrial function, reduce oxidative stress, and facilitate autophagic clearance of toxic protein aggregates. However, excessive or prolonged AMPK activation has also been implicated in tau hyperphosphorylation and neuronal injury, suggesting that the effects of AMPK are highly context-dependent and vary with disease stage, cell type, and the duration of activation [86].

Emerging evidence indicates that AMPK also participates in the pathophysiology of Parkinson’s disease (PD). Mitochondrial dysfunction, impaired proteostasis, oxidative stress, and accumulation of α-synuclein contribute to progressive dopaminergic neurodegeneration. Given the established roles of AMPK in mitochondrial homeostasis and autophagy, appropriate AMPK activation may facilitate neuronal adaptation to energetic and proteotoxic stress [1]. Consistent with this concept, recent experimental evidence demonstrates that pharmacological AMPK activation reduces oxidative stress and phosphorylated α-synuclein accumulation, enhances mitophagy and lysosomal degradation, improves mitochondrial biogenesis, and attenuates dopaminergic degeneration in cellular and mouse models of PD [87]. These findings support AMPK as a potential therapeutic target in PD, although its effects are likely to depend on the magnitude, duration, and cellular context of activation.

Glaucoma is increasingly recognized as a neurodegenerative disorder characterized by progressive retinal ganglion cell (RGC) and optic nerve degeneration, in which impaired energy metabolism, mitochondrial dysfunction, oxidative stress, and defective mitochondrial quality control contribute to neuronal vulnerability [88,89]. Emerging evidence also implicates AMPK signaling in RGC survival. Experimental AMPK activation has been reported to attenuate RGC apoptosis through suppression of mTOR signaling [90]. However, other studies have observed RGC protection in association with suppression of stress-induced AMPK activation, suggesting that the role of AMPK in retinal neurodegeneration is context-dependent [91]. Thus, rather than supporting indiscriminate AMPK activation, current evidence suggests that precise modulation of AMPK signaling may represent a potential neuroprotective strategy in glaucoma.

### 4.5. Aging

Aging is characterized by progressive loss of cellular and tissue homeostasis and increased susceptibility to metabolic disease, cardiovascular disorders, neurodegeneration, cancer, sarcopenia, and frailty. Major hallmarks of aging include mitochondrial dysfunction, loss of proteostasis, impaired autophagy, genomic instability, chronic inflammation, stem-cell exhaustion, and deregulated nutrient sensing [92,93]. AMPK intersects with many of these processes and represents an important link between cellular energy status and pathways regulating healthspan and longevity. Although AMPK activity is not uniformly reduced in all aged tissues or physiological settings, several studies indicate that the capacity for AMPK activation declines with aging, potentially compromising metabolic adaptation, autophagy, mitochondrial quality control, and stress resistance [94]. This age-related impairment appears to be tissue- and context-dependent and may involve increased phosphatase activity and inhibitory upstream signaling [94].

AMPK is closely integrated with several evolutionarily conserved longevity pathways, including mTOR, sirtuins, FOXO transcription factors, and autophagy. AMPK suppresses mTORC1 signaling through phosphorylation of tuberous sclerosis complex 2 (TSC2) and Raptor, thereby shifting cellular metabolism away from anabolic growth toward maintenance and stress adaptation [95]. AMPK also promotes autophagy directly through phosphorylation of ULK1 and indirectly through inhibition of mTORC1, facilitating the clearance of damaged proteins and dysfunctional mitochondria and thereby preserving cellular proteostasis [96]. In parallel, AMPK interacts with the SIRT1–PGC-1α–FOXO network. AMPK increases cellular NAD^+^ availability and enhances SIRT1 activity, leading to deacetylation and regulation of PGC-1α, FOXO1, and FOXO3 and promoting mitochondrial biogenesis, oxidative metabolism, antioxidant defense, and stress resistance [41,97]. Through these coordinated pathways, AMPK links nutrient and energy sensing with cellular programs that support maintenance and resilience during aging.

Evidence from model organisms further supports a role for AMPK in lifespan and healthspan regulation. In *Caenorhabditis elegans*, the AMPK catalytic α-subunit AAK-2 links cellular energy status to insulin-like signaling and is required for lifespan extension produced by several energetic and longevity-promoting interventions; increased AAK-2 activity itself can also promote longevity [98]. These findings establish AMPK as an evolutionarily conserved component of nutrient-sensing longevity networks. In mammals, interventions associated with improved healthspan, including caloric restriction, intermittent fasting, and exercise, engage AMPK together with other nutrient-sensing pathways and promote mitochondrial remodeling, fatty acid oxidation, autophagy, and metabolic adaptation [99,100]. Long-term low-dose metformin treatment has also been reported to improve healthspan and extend lifespan in male mice, whereas a substantially higher dose was toxic, illustrating the importance of dose and therapeutic window in metabolic geroprotection [101]. More recently, the compartment-selective AMPK activator aldometanib was reported to extend lifespan and healthspan in *C. elegans* and mice while improving metabolic homeostasis [22].

These observations have stimulated considerable interest in AMPK activation as a potential gerotherapeutic strategy. Metformin is being investigated extensively in the context of aging because of its effects on cellular energetics, nutrient sensing, inflammation, and metabolic homeostasis [102]. However, the potential benefits of AMPK activation must be balanced against the risks of sustained or indiscriminate pathway activation. AMPK functions are strongly tissue-, cell-, and context-dependent, and chronic activation can interfere with physiological processes requiring anabolic metabolism. For example, the systemic pan-AMPK activator MK-8722 produced robust improvements in glucose homeostasis in rodents and non-human primates but also caused cardiac glycogen accumulation and cardiac hypertrophy during chronic treatment [103]. In addition, AMPK may support survival and metabolic adaptation of established tumor cells under energetic stress, while sustained activation in pancreatic β-cells or hypothalamic circuits may adversely affect insulin secretion or whole-body energy balance in selected contexts. Thus, the therapeutic objective in aging should not be continuous systemic AMPK activation, but rather restoration or selective enhancement of appropriate AMPK signaling in disease-relevant tissues, cellular populations, and subcellular compartments.

Collectively, AMPK occupies a central position within the nutrient-sensing networks that regulate aging through coordinated effects on mTORC1, SIRT1/FOXO signaling, autophagy, mitochondrial quality control, inflammation, and cellular stress resistance. Genetic and pharmacological studies in model organisms support a contribution of AMPK signaling to lifespan and healthspan extension, but whether direct AMPK activation can safely extend human healthspan or lifespan remains unknown. Future gerotherapeutic strategies will therefore likely require careful optimization of the magnitude, duration, tissue distribution, isoform selectivity, and subcellular localization of AMPK activation. Tissue-, isoform-, and compartment-selective modulation may preserve the beneficial effects of AMPK on cellular maintenance while minimizing adverse consequences associated with chronic systemic activation.

## 5. Therapeutic Targeting of AMPK

### 5.1. Indirect AMPK Activators: Metformin and Related Agents

Indirect activation of AMPK is an established strategy for exploiting its therapeutic potential. Unlike direct agonists that bind the AMPK heterotrimeric complex, indirect activators stimulate AMPK by perturbing cellular energy homeostasis, most commonly through inhibition of mitochondrial ATP production or modulation of upstream metabolic signaling pathways (Table 4). The resulting increase in intracellular AMP and ADP concentrations promotes phosphorylation of the catalytic α-subunit at Thr172 by upstream kinases, primarily liver kinase B1 (LKB1), thereby initiating an adaptive response that restores cellular energy balance and metabolic homeostasis [1,3,6,46,50]. In addition to regulating glucose and lipid metabolism, AMPK activation suppresses anabolic processes while promoting catabolic pathways, autophagy, and mitochondrial quality control, making indirect activation an attractive therapeutic strategy for a wide range of cardiometabolic disorders [2,46,50,104].

Metformin is a first-line pharmacological therapy for T2DM that can activate AMPK through dose- and compartment-dependent mechanisms. At higher or metabolically effective exposures, metformin inhibits mitochondrial respiratory chain complex I, reducing mitochondrial ATP production and altering cellular adenine nucleotide ratios, thereby favoring LKB1-dependent AMPK activation [105,106,107,108]. Recent in vivo evidence further supports mitochondrial complex I as an important acute molecular target through which metformin lowers blood glucose [106]. In parallel, low-dose metformin can activate the lysosome-associated AMPK pathway without producing substantial changes in whole-cell adenine nucleotide levels. In this pathway, metformin binds presenilin enhancer protein 2 (PEN2), which engages the v-ATPase accessory subunit ATP6AP1 and intersects with the lysosomal glucose-sensing machinery to activate AMPK [109]. Thus, mitochondrial energetic stress and lysosomal nutrient-sensing mechanisms may contribute to AMPK activation in a dose-, tissue-, and subcellular compartment-dependent manner. Although early studies attributed the glucose-lowering effects of metformin partly to AMPK-dependent suppression of hepatic gluconeogenesis, subsequent genetic studies demonstrated that metformin can inhibit hepatic glucose production independently of hepatic LKB1–AMPK signaling [110]. AMPK is therefore not considered fundamentally required for the acute suppression of hepatic gluconeogenesis by metformin. Nevertheless, AMPK contributes to important chronic metabolic actions of metformin, particularly through phosphorylation and inhibition of ACC1 and ACC2, resulting in suppression of *de novo* lipogenesis and modulation of fatty acid metabolism. Genetic disruption of AMPK-mediated ACC phosphorylation impairs the lipid-lowering and insulin-sensitizing effects of metformin, supporting an important role for the AMPK–ACC axis in chronic metabolic regulation [71]. These effects can reduce hepatic lipid accumulation and improve insulin sensitivity, thereby indirectly contributing to improved hepatic glucose homeostasis during chronic treatment.

Similarly, metformin appears to activate lysosome-associated AMPK independently of changes in cellular adenine nucleotide levels [109]. In relation to this, intestine-specific actions, alterations in cellular redox state, modulation of glucagon signaling, and remodeling of the gut microbiota contribute to metformin’s metabolic efficacy [60,65,111,112]. Consequently, AMPK-dependent and AMPK-independent pathways cooperate to mediate the therapeutic actions of metformin, with the relative contribution of each pathway varying among tissues, drug concentrations, and disease states [65,109,110,111].

Natural products constitute an important and mechanistically diverse class of AMPK modulators. Several widely investigated metabolic agents are either naturally occurring compounds or were historically inspired by natural products. In contrast to highly optimized synthetic direct AMPK agonists, most natural products activate AMPK indirectly by altering cellular energy status, mitochondrial function, Ca^2+^ signaling, redox balance, or upstream regulatory pathways. Consequently, their pharmacological effects are frequently pleiotropic and cannot be attributed exclusively to AMPK activation.

Berberine—an isoquinoline alkaloid found in medicinal plants including *Coptis* and *Berberis* species—is one of the best-characterized natural AMPK activators. Berberine increases AMPK activity and improves glucose and lipid metabolism in experimental models [113]. Mechanistically, berberine inhibits mitochondrial respiratory complex I, producing mild energetic stress and increasing cellular AMP/ATP ratios, thereby activating AMPK [114]. Berberine has also been reported to maintain AMPK activity through lysosomal mechanisms involving inhibition of the AMPK dephosphorylation regulator UHRF1 [115]. However, the metabolic effects of berberine are not exclusively AMPK-dependent, consistent with its broad polypharmacology and effects on glycolysis, gut microbiota, inflammatory pathways, and lipid metabolism. A major translational limitation is its very low oral bioavailability, which has been attributed to poor solubility and permeability, intestinal efflux, and extensive intestinal and hepatic metabolism. Recent reviews estimate systemic oral bioavailability at below 1% [116].

Resveratrol, a stilbene polyphenol present in grapes, berries, and peanuts, also activates AMPK indirectly. Resveratrol-dependent AMPK activation is closely linked to SIRT1 signaling and mitochondrial regulation [117]. Price et al. demonstrated that SIRT1 is required for AMPK activation and for several beneficial mitochondrial effects of resveratrol [117]. Other experimental studies suggest that Ca^2+^–CaMKKβ signaling can also participate in resveratrol-mediated AMPK activation in selected cellular contexts [118]. Resveratrol has undergone extensive preclinical and human investigation, but its clinical translation is limited by rapid intestinal and hepatic metabolism; despite substantial absorption, the oral bioavailability of unchanged resveratrol is considerably below 1% [119]. Thus, its biological effects likely reflect combined actions on AMPK, SIRT1, redox signaling, inflammation, and additional molecular pathways.

Ginsenosides, the major bioactive saponins of *Panax ginseng*, represent another group of natural compounds that influence AMPK signaling. Multiple ginsenosides, including Rg1, Rg3, Rb-family compounds, Rh-family compounds, and the intestinal microbial metabolite compound K, have been reported to regulate AMPK-dependent metabolic pathways [120]. Compound K reduces hepatic lipid accumulation through AMPK activation, whereas ginsenoside Rg3 increases AMPK activity and reduces hepatic triglyceride and cholesterol accumulation in experimental models [121,122]. However, the upstream mechanisms of AMPK activation vary among individual ginsenosides and remain incompletely defined. Their pharmacology is further complicated by poor and variable intestinal absorption and dependence on gut microbial metabolism, making systemic exposure and pharmacodynamic responses highly variable.

Salicylate provides an important contrast to most natural indirect activators because it can directly activate AMPK. Salicylate binds preferentially to β1-containing AMPK complexes at an allosteric regulatory region overlapping the ADaM site, promoting allosteric activation and protecting Thr172 from dephosphorylation [123]. Thus, salicylate activates AMPK independently of increases in AMP or ADP. Nevertheless, salicylate and its clinically used derivatives also inhibit cyclooxygenases and engage multiple inflammatory pathways, illustrating that even a natural compound capable of direct AMPK activation remains pharmacologically pleiotropic.

Natural-product AMPK activators therefore present both opportunities and substantial translational challenges. Poor bioavailability is common among compounds such as berberine, resveratrol, and several ginsenosides, limiting predictable exposure in target tissues. Polypharmacology further complicates mechanistic interpretation because these compounds frequently regulate mitochondrial function, redox signaling, inflammatory pathways, nuclear receptors, enzymes, and the gut microbiome in addition to AMPK. Moreover, botanical preparations introduce an additional challenge of batch-to-batch variability, because plant genotype, cultivation conditions, harvest time, extraction procedures, and processing can alter chemical composition. Regulatory guidance for botanical drug development therefore emphasizes chemical characterization, raw-material control, and demonstration of batch-to-batch therapeutic consistency.

Natural indirect activators and synthetic direct AMPK agonists also differ in their therapeutic windows. Natural compounds generally produce relatively modest and context-dependent AMPK activation because their effects depend on cellular uptake, metabolism, energetic state, and engagement of upstream signaling pathways. Such moderate activation may reduce the risk of sustained pan-AMPK stimulation but is accompanied by variable target engagement and extensive off-target pharmacology. In contrast, synthetic direct activators, such as compound 991, PF-739, and MK-8722, provide more predictable and potent AMPK activation and can be engineered for isoform selectivity. However, potent systemic activation may narrow the therapeutic window by engaging AMPK complexes in tissues in which prolonged activation is undesirable, as illustrated by cardiac glycogen accumulation and hypertrophy with MK-8722. Thus, the apparent broader therapeutic window of some natural activators should not necessarily be interpreted as greater AMPK selectivity; rather, it may reflect weaker target engagement, lower systemic exposure, and distributed polypharmacology. Future development may benefit from combining the relatively mild physiological modulation characteristic of natural activators with the tissue-, isoform-, and compartment-selectivity achievable with synthetic AMPK agonists.

In addition to pharmacological agents, accumulating evidence indicates that endogenous and gut microbiota-derived metabolites contribute to physiological AMPK regulation. Short-chain fatty acids (SCFAs), particularly acetate, propionate, and butyrate, can stimulate AMPK signaling in several cellular and tissue contexts. For example, acetate, propionate, and butyrate increase AMPK phosphorylation in intestinal epithelial cells, whereas hepatic propionate signaling can activate AMPK through a GPR43–Ca^2+^–CaMKKβ-dependent pathway [124,125]. These observations suggest that microbiota-derived SCFAs can couple nutrient and microbial signals to host AMPK activity. In addition, long-chain fatty acyl-CoA esters have been identified as endogenous AMPK activators that directly bind the allosteric drug and metabolite (ADaM) site and preferentially activate β1-containing AMPK complexes [126]. More recently, lithocholic acid (LCA), a gut microbiota-derived secondary bile acid whose circulating levels increase during caloric restriction, has emerged as an endogenous regulator of AMPK [127,128]. At physiologically relevant concentrations, LCA activates AMPK independently of substantial changes in cellular adenine nucleotide ratios and recapitulates several beneficial effects of caloric restriction, including improved skeletal muscle function and healthy aging [127]. Mechanistically, LCA binds TULP3 and activates sirtuins, leading to regulation of v-ATPase and subsequent AMPK activation through the lysosomal glucose-sensing pathway [128]. Collectively, these findings demonstrate that endogenous and microbiota-derived metabolites can regulate AMPK through multiple mechanisms and highlight the therapeutic potential of harnessing physiological metabolite-sensing pathways to modulate AMPK signaling.

Exercise is one of the most potent physiological activators of AMPK, particularly in skeletal muscle, where transient ATP depletion stimulates glucose uptake, fatty acid oxidation, and mitochondrial biogenesis and autophagy [129]. Similarly, caloric restriction and intermittent fasting activate AMPK in multiple metabolic tissues, contributing to improved insulin sensitivity, enhanced metabolic flexibility, and reduced chronic inflammation [130,131]. The substantial overlap between exercise-induced signaling and pharmacological AMPK activation has stimulated the development of exercise mimetics that aim to reproduce the metabolic benefits of physical activity in individuals unable to exercise adequately [132].

Despite clinical efficacy, indirect AMPK activators have limitations. Because they rely on alterations in cellular energetic status rather than direct engagement of the AMPK complex, their pharmacological effects are highly influenced by tissue metabolic state, mitochondrial function, and intracellular drug accumulation [65,104,110,111]. Furthermore, many indirect activators exhibit pleiotropic biological activities, making it difficult to distinguish AMPK-dependent from AMPK-independent effects in both experimental and clinical settings [65,110,111,133]. Chronic perturbation of mitochondrial respiration may also increase the risk of off-target toxicity in susceptible tissues, while the lack of isoform and tissue selectivity may limit therapeutic precision [134,135]. These limitations have driven the development of direct small-molecule AMPK activators that engage defined structural sites within the AMPK complex to achieve greater potency, selectivity, and translational potential.

### 5.2. Direct Small-Molecule AMPK Activators

Direct activating molecules bind directly to the AMPK heterotrimer, producing rapid and selective activation. Most direct activators target the allosteric drug and metabolite (ADaM) site, a binding pocket located at the interface between the kinase domain of the α-subunit and the carbohydrate-binding module of the β-subunit. Binding to the ADaM site allosterically enhances kinase activity while simultaneously protecting Thr172 from dephosphorylation, thereby sustaining AMPK activation and downstream metabolic signaling [6,104,134,136,137]. This mechanism provides greater pharmacological specificity than indirect activation and offers improved opportunities for rational drug design.

The direct AMPK activator A-769662 provided proof of concept that pharmacological activation of the ADaM site could improve metabolic homeostasis. A-769662 preferentially activates β1-containing AMPK complexes, stimulates fatty acid oxidation, suppresses hepatic lipogenesis, and enhances glucose uptake in skeletal muscle [135,136,138]. However, its limited oral bioavailability and relatively modest potency have restricted clinical development. Subsequent medicinal chemistry efforts generated more potent ADaM-site agonists, including Compound 991 (EX229), PF-739, PF-249, and Compound 13, which exhibited improved pharmacokinetic properties and more robust activation of AMPK signaling [15,129,137,139,140].

Among next-generation activators, MK-8722 functions as a pan-activator capable of activating all twelve mammalian AMPK heterotrimeric complexes. In rodents and non-human primates, systemic administration of MK-8722 markedly improved glucose homeostasis, enhanced insulin-independent glucose uptake, and reduced hyperglycemia without increasing the risk of hypoglycemia [129,141]. However, chronic treatment induced reversible cardiac glycogen accumulation and cardiac hypertrophy, highlighting issues with sustained systemic AMPK activation [129].

To bypass these unwanted side effects, isoform-selective and tissue-targeted AMPK activators are in development. For example, PF-739 preferentially activates γ1-containing complexes in skeletal muscle and effectively lowers blood glucose by increasing muscle glucose uptake while minimizing activation of γ3-containing complexes [139]. Likewise, β1-selective activators exploit differences in the ADAM-site architecture between β1 and β2 isoforms, selectively targeting liver or macrophage AMPK while reducing unwanted effects in cardiac and skeletal muscle [23,134]. Advances in cryo-electron microscopy and structure-guided drug design have identified novel ligands capable of stabilizing specific AMPK conformations, providing a rational framework for developing next-generation precision therapeutics [16,134].

Beyond ADaM-site agonists, recent studies have highlighted the therapeutic potential of targeting spatially distinct pools of AMPK. AMPK activation is compartmentalized within cells, with lysosomal AMPK responding to relatively mild glucose deprivation before broader activation of cytosolic and mitochondrial AMPK pools under more severe energetic stress [18,20]. Aldometanib (LXY-05-029), a small-molecule aldolase inhibitor, selectively activates lysosomal AMPK by preventing fructose-1,6-bisphosphate (FBP) from binding to lysosome-associated aldolase, thereby initiating the v-ATPase–Ragulator–AXIN/LKB1 signaling pathway and mimicking cellular glucose starvation without substantially altering adenine nucleotide ratios [22]. In preclinical models, aldometanib inhibited hepatic de novo lipogenesis, improved glucose homeostasis and insulin sensitivity, and alleviated fatty liver and steatohepatitis in obese rodents [22]. Aldometanib also extended lifespan and healthspan in *C. elegans* and mice without producing detectable cardiac hypertrophy or dysfunction in treated mice [22]. These findings suggest that selective engagement of compartmentalized AMPK signaling may provide an alternative to widespread AMPK activation and support further development of spatially targeted approaches for metabolic and age-related diseases [18,22].

Despite these advances, challenges remain before direct AMPK activators can be translated into routine clinical practice. Long-term systemic activation may disrupt tissue-specific metabolic programs, particularly in the heart, where excessive glycogen accumulation and cardiac hypertrophy have emerged as recurring adverse effects in preclinical studies of pan-AMPK activators such as MK-8722 [103]. Beyond the cardiovascular system, sustained AMPK activation may also produce undesirable effects in other tissues depending on the physiological context. For example, AMPK participates in hypothalamic regulation of food intake and whole-body energy balance, whereas forced or sustained AMPK activation in pancreatic β-cells has been reported to suppress glucose-stimulated insulin secretion in experimental models, illustrating that AMPK activation is not universally beneficial across all tissues and physiological contexts [142]. O304, a pharmacological pan-AMPK activator, progressed to a Phase IIa proof-of-concept study in metformin-treated patients with T2D. O304 was associated with improvements in fasting plasma glucose, insulin resistance, blood pressure, and microvascular perfusion; however, the magnitude of clinical efficacy was more modest than the pronounced metabolic responses observed in experimental models [143]. Furthermore, although AMPK has traditionally been regarded as a tumor suppressor because of its ability to restrain anabolic metabolism and cell growth, accumulating evidence demonstrates that AMPK can also promote tumor-cell survival under metabolic stress. AMPK supports cellular redox and energy homeostasis, promotes mitochondrial metabolic adaptation, and enables cancer cells to tolerate nutrient deprivation and other stresses [76,84,144]. Accordingly, AMPK signaling can support tumor growth [76,84,145] and persistence in selected cancer contexts, emphasizing its context-dependent role in cancer biology [76,84,144]. These findings indicate that the therapeutic consequences of AMPK activation are determined not only by the magnitude and duration of activation but also by tissue specificity, disease stage, and metabolic context.

Safety represents an additional challenge for chronic pan-AMPK activation. MK-8722 is a potent systemic activator capable of activating all twelve mammalian AMPK heterotrimers and produced substantial improvements in glucose homeostasis in rodents and non-human primates. However, chronic treatment caused reversible cardiac glycogen accumulation and cardiac hypertrophy [103]. This phenotype demonstrates that sustained activation of AMPK can produce tissue-specific adverse effects even when systemic metabolic outcomes are favorable. Increased myocardial glucose uptake and glycogen synthesis may contribute to this cardiac phenotype, although the magnitude of toxicity is likely to depend on compound exposure, duration of treatment, and the AMPK complexes engaged. Importantly, cardiac hypertrophy does not appear to be an inevitable consequence of all pharmacological AMPK activation; O304 increased cardiac AMPK activity and glucose uptake in experimental models without increasing heart weight, illustrating that cardiovascular outcomes can differ substantially among AMPK activators [143].

Isoform- and complex-selective AMPK activation therefore represents a promising strategy to improve the therapeutic index of direct agonists. Because the composition of AMPK heterotrimers differs among tissues, structural differences within the ADaM site can be exploited to preferentially activate disease-relevant complexes while limiting AMPK activation in tissues in which sustained signaling may be undesirable. For example, the β1-selective activator PF-249 activates hepatic β1-containing AMPK complexes but does not effectively activate skeletal-muscle AMPK, whereas PF-739 activates a broader range of AMPK complexes and produces AMPK-dependent skeletal-muscle glucose disposal [140]. Similarly, SC4 preferentially activates α2-containing AMPK complexes and potently activates the α2β2γ1 complex that is prominent in skeletal muscle, providing a structural proof of concept for the development of β2- and muscle-directed AMPK therapeutics [145]. These findings suggest that the objective of future AMPK drug development should not be maximal pan-AMPK activation, but rather optimization of the magnitude, duration, tissue distribution, heterotrimer selectivity, and subcellular localization of AMPK signaling. Such precision approaches may preserve the metabolic benefits of AMPK activation while reducing the cardiac and other adverse effects associated with chronic systemic activation.

### 5.3. Tissue-Specific Modulation of AMPK Signaling

**Figure 3:**
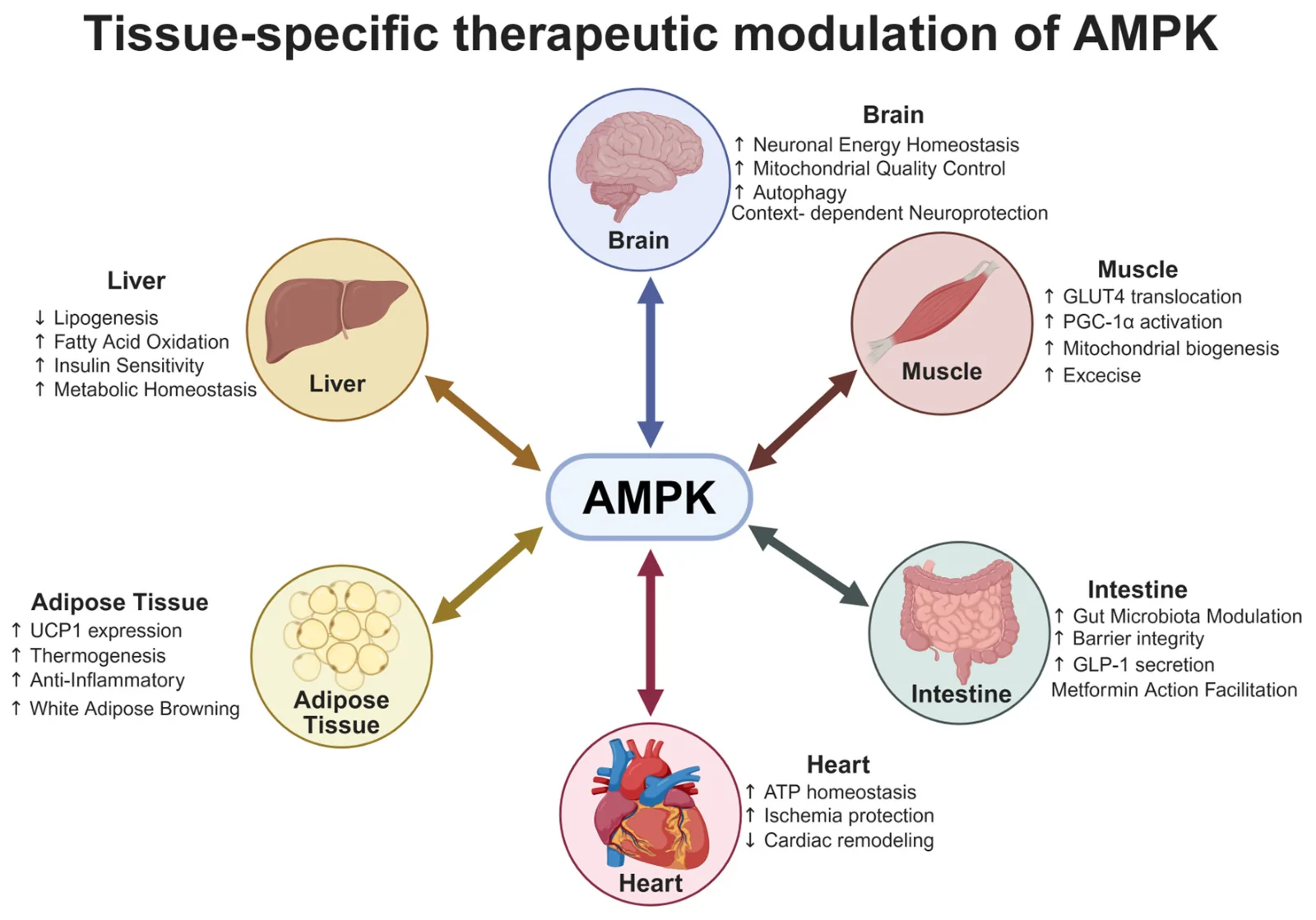
Tissue-Specific Therapeutic Modulation of AMPK. AMPK functions as a master metabolic sensor and is expressed in many tissues and organs, which are involved in restoring energy homeostasis. AMPK has distinct physiological effects across the brain, muscle, liver, intestine, heart, and adipose tissue. AMPK tissue-specific responses result in physiological functions that vary among tissues. This reality supports targeting tissue-specific AMPK. Bidirectional arrows between AMPK and individual tissues indicate tissue-specific interactions and modulation of AMPK signaling. Upward arrows (↑) indicate increased or enhanced biological processes, whereas downward arrows (↓) indicate decreased or suppressed processes. Created in BioRender. Ceballos, H. (2026) imfd87e https://BioRender.com.

AMPK is ubiquitously expressed. However, its physiological functions vary considerably among tissues owing to differences in heterotrimeric isoform composition, metabolic demands, and downstream signaling networks [145,146,147]. Consequently, therapeutic activation of AMPK is unlikely to produce uniform biological responses across organs, making tissue-specific modulation an increasingly important objective in drug development (Figure 3).

The liver is one of the primary target organs for AMPK-directed therapies. Hepatic AMPK functions as a master metabolic switch that coordinates the transition from anabolic to catabolic metabolism during energetic stress. Although early studies suggested that AMPK activation directly suppresses hepatic gluconeogenesis through regulation of transcriptional pathways involving CRTC2 and class IIa histone deacetylases [148,149,150], subsequent genetic and pharmacological studies have challenged the requirement for hepatic AMPK in the acute suppression of hepatic glucose production. In particular, metformin-mediated inhibition of hepatic gluconeogenesis is preserved in the absence of hepatic LKB1–AMPK signaling [110], and genetic studies indicate that hepatic AMPK is not fundamentally required for the acute suppression of hepatic gluconeogenesis. Moreover, the direct AMPK activator PF-739 lowers blood glucose primarily by enhancing skeletal muscle glucose disposal without significantly reducing hepatic glucose production [140]. Thus, AMPK activation does not appear to be fundamentally required for the acute inhibition of hepatic gluconeogenesis [110,133,140,151,152,153]. Instead, a well-established function of hepatic AMPK is the regulation of lipid metabolism. AMPK phosphorylates and inhibits acetyl-CoA carboxylase (ACC), thereby reducing malonyl-CoA levels, suppressing *de novo* lipogenesis, and promoting fatty acid oxidation. AMPK also inhibits SREBP-1c-dependent lipogenic programs, thereby reducing the expression of lipogenic enzymes, including fatty acid synthase (FASN), stearoyl-CoA desaturase-1 (SCD1), and ACC1 [154]. These actions reduce hepatic triglyceride accumulation and improve hepatic insulin sensitivity, suggesting that AMPK may contribute to the chronic, indirect suppression of hepatic glucose production by alleviating hepatic steatosis and insulin resistance [16,62,155,156]. Hepatic AMPK also regulates cholesterol biosynthesis through phosphorylation of 3-hydroxy-3-methylglutaryl-CoA reductase (HMGCR), stimulates autophagy and mitochondrial quality control, and attenuates oxidative stress and inflammatory signaling, thereby limiting progression from simple steatosis to steatohepatitis and fibrosis [6,46,62,155,156]. Collectively, these findings indicate that the therapeutic benefits of hepatic AMPK activation are more strongly supported by its effects on lipid metabolism, hepatic steatosis, and insulin sensitivity than by a direct requirement for AMPK in suppressing hepatic gluconeogenesis. Consequently, therapeutic strategies are expected to focus on liver-selective AMPK activation or precision modulation of downstream signaling networks to maximize metabolic benefits.

Adipose tissue is an attractive therapeutic target for AMPK-directed interventions. Activation of AMPK suppresses lipogenesis by phosphorylating ACC and inhibiting SREBP-1c while promoting fatty acid β-oxidation through activation of carnitine palmitoyltransferase-1 (CPT1) and mitochondrial oxidative metabolism [1,2,6,62,157]. In addition to regulating lipid turnover, AMPK promotes mitochondrial biogenesis via the PGC-1α signaling pathway and stimulates the browning of white adipocytes by increasing the expression of thermogenic genes, including uncoupling protein 1, PRDM16, and PPARγ coactivator-1α, thereby enhancing adaptive thermogenesis and whole-body energy expenditure [6,62,131,158,159]. Adipocyte AMPK modulates adipose tissue inflammation by suppressing NF-κB signaling and promoting macrophage polarization toward an anti-inflammatory phenotype, reducing chronic low-grade inflammation associated with obesity and insulin resistance [160,161,162]. Collectively, these findings establish adipose AMPK as a multifaceted regulator of metabolic homeostasis that integrates lipid metabolism, thermogenesis and immune responses. Consequently, therapeutic strategies aimed at selectively activating adipose AMPK may provide substantial benefits for the treatment of obesity, T2D, and metabolic dysfunction-associated steatotic liver disease (MASLD), while minimizing the adverse effects associated with systemic AMPK activation.

Skeletal muscle is the largest insulin-sensitive organ because it accounts for the majority of whole-body glucose disposal during both exercise and postprandial metabolism. AMPK functions as a cellular energy sensor that is rapidly activated during muscle contraction, hypoxia, and nutrient deprivation, coordinating metabolic adaptation to energetic stress. Upon activation, AMPK stimulates glucose uptake through insulin-independent translocation of GLUT4, enhances fatty acid β-oxidation by phosphorylating ACC, and promotes mitochondrial biogenesis through activation of PGC-1α, thereby improving oxidative capacity and metabolic flexibility [2,62,163,164,165]. In addition, AMPK activates autophagy through phosphorylation of ULK1 and suppresses mTORC1, facilitating mitochondrial quality control and adaptation to prolonged exercise and metabolic stress [2,46,166]. Consequently, AMPK accounts for the metabolic benefits of exercise and is an attractive target for the development of exercise mimetics [6,132]. Direct AMPK activators, including PF-739, increase skeletal muscle glucose uptake and lower blood glucose concentrations in rodents and primates independent of insulin signaling [139,140]. However, distinct AMPK heterotrimeric complexes exhibit fiber type-specific expression and physiological functions, with α2β2γ3 complexes being dominant in contraction-induced glucose uptake in glycolytic muscle. This emphasizes that isoform-selective and muscle-targeted AMPK activation can maximize metabolic efficacy while limiting systemic adverse effects [13,17,134,163]. In addition to its metabolic functions, AMPK has emerged as an important regulator of skeletal muscle health and disease. Altered energy metabolism, mitochondrial dysfunction, and defective protein and organelle quality-control pathways are common features of neuromuscular disorders, including Duchenne muscular dystrophy (DMD), spinal muscular atrophy (SMA), myotonic dystrophy type 1 (DM1), and age-related sarcopenia [163,167,168,169]. AMPK regulates several processes relevant to muscle maintenance, including mitochondrial biogenesis and quality control, autophagy, substrate oxidation, and muscle protein turnover [163,167,169]. Experimental studies, particularly in DMD models, indicate that AMPK activation can enhance oxidative metabolism and mitochondrial function, promote autophagy, and attenuate dystrophic pathology, supporting further investigation of AMPK-directed approaches for neuromuscular and age-related muscle disorders [163,167,168,169].

The intestinal epithelium is an important tissue-specific target of AMPK signaling, extending the therapeutic relevance of AMPK beyond its metabolic functions in the liver, skeletal muscle, and adipose tissue. Intestinal AMPK maintains epithelial energy homeostasis, preserves barrier integrity, and coordinates host–microbiota interactions. Activation of AMPK promotes tight junction assembly through regulation of junctional proteins such as occludin, claudins, and ZO-1, thereby limiting intestinal permeability and preventing the translocation of microbial products that drive systemic inflammation [57,65,111,170]. In addition, AMPK regulates intestinal epithelial cell proliferation, autophagy, and mitochondrial quality control, processes that are essential for epithelial renewal and mucosal repair [46,57]. Intestinal AMPK functions as an endocrine and microbial signaling hub capable of communicating with peripheral metabolic tissues. Intestine-specific loss of AMPKα1 demonstrated changes in antimicrobial peptide expression, gut microbiota composition and microbial metabolites, and brown adipose tissue thermogenesis through an intestine–BAT signaling axis. Importantly, intestinal AMPK deficiency abolished much of the metabolic benefit of metformin, indicating that intestinal AMPK is an essential mediator of metformin-induced improvements in energy expenditure and glucose homeostasis [60]. Together with its roles in nutrient sensing, GLP-1 secretion, and maintenance of epithelial barrier function, selective activation of intestinal AMPK represents a promising therapeutic strategy for obesity, T2D, MASLD, and other chronic metabolic disorders [65,109,171,172].

AMPK signaling within the central nervous system (CNS) is highly region-, cell type-, and isoform-dependent and exerts important effects on both neuronal metabolism and whole-body energy homeostasis. Both AMPKα1- and AMPKα2-containing complexes are expressed in the CNS, but their physiological functions differ among neuronal populations and brain regions. The hypothalamus is particularly important because AMPK integrates nutrient, hormonal, and neuronal signals within the arcuate nucleus (ARC), ventromedial hypothalamus (VMH), dorsomedial hypothalamus, and other metabolically responsive nuclei to regulate food intake, glucose homeostasis, thermogenesis, and whole-body energy expenditure [173,174]. Genetic studies have demonstrated that AMPKα2 has distinct functions in proopiomelanocortin (POMC) and agouti-related peptide (AgRP) neurons; deletion of AMPKα2 in these neuronal populations produces different alterations in feeding, glucose sensing, and energy balance, illustrating the cell-specific nature of hypothalamic AMPK signaling [175]. More recent evidence further demonstrates specialization among regulatory AMPK subunits. AMPKγ2 is highly enriched in steroidogenic factor-1 (SF1) neurons of the VMH, and selective loss of AMPKγ2 in these neurons decreases brown adipose tissue thermogenesis and promotes obesity, highlighting the functional importance of AMPK heterotrimer composition within specific hypothalamic circuits [176]. Thus, CNS AMPK should not be considered a uniform signaling pool; rather, its metabolic actions depend on brain region, neuronal population, and heterotrimeric composition.

Beyond hypothalamic regulation of energy balance, AMPK plays context-dependent roles in neuronal survival, glial metabolism, and neuroinflammation. Under moderate energetic or oxidative stress, AMPK can support neuronal homeostasis by promoting mitochondrial quality control, autophagy, and adaptive energy metabolism. AMPK activity in astrocytes is also important for neuronal survival: astrocyte-specific AMPK deficiency disrupts glucose uptake and lactate production through altered TXNIP–GLUT1 signaling, thereby impairing the astrocyte–neuron lactate shuttle and causing neuronal loss [177]. AMPK additionally modulates neuroimmune responses in microglia. Experimental activation of AMPK suppresses pro-inflammatory microglial signaling and reduces inflammatory mediators, in part through inhibition of NF-κB-associated pathways and modulation of microglial polarization [178]. These mechanisms are potentially relevant to neurodegenerative disorders characterized by mitochondrial dysfunction, impaired proteostasis, oxidative stress, and chronic neuroinflammation. However, AMPK activation is not uniformly neuroprotective. Sustained or excessive neuronal AMPK activation can induce excessive autophagy and synaptic loss, indicating that the effects of AMPK depend on the magnitude and duration of activation and the cellular and disease context [179]. Thus, restoration of physiological AMPK signaling may be beneficial in some neurological settings, whereas prolonged or indiscriminate activation could exacerbate neuronal dysfunction.

Therapeutic targeting of CNS AMPK therefore presents challenges that differ substantially from those encountered in peripheral tissues. The blood–brain barrier (BBB) restricts the entry of many pharmacological agents into the brain, making CNS exposure strongly dependent on molecular size, lipophilicity, transporter interactions, and other pharmacokinetic properties. At the same time, systemic AMPK activators may engage peripheral AMPK complexes and CNS AMPK pools with different or even opposing physiological effects. This issue is particularly relevant for metabolic diseases because AMPK activation in skeletal muscle, liver, or adipose tissue can improve aspects of metabolic homeostasis, whereas sustained hypothalamic AMPK activation may stimulate feeding and reduce thermogenesis [174]. Consequently, CNS-directed AMPK therapies will require careful control of BBB penetration, dose, duration, and cellular selectivity. Brain-region-, cell-type-, isoform-, and compartment-selective approaches may be particularly valuable for neurodegenerative diseases, whereas peripherally restricted AMPK activators may be preferable for metabolic indications in which hypothalamic AMPK activation could counteract the desired therapeutic effect. These considerations further support the development of precision AMPK modulators rather than indiscriminate systemic activation.

Collectively, these findings support a shift from systemic AMPK activation toward tissue-specific therapeutic modulation. Emerging strategies, including isoform-selective activators, targeted nanoparticle delivery, tissue-specific prodrugs, and gene-editing technologies [53,145,180,181], may enable selective activation of disease-relevant AMPK complexes while minimizing off-target effects.

## 6. Perspectives

Herein, the different aspects of the AMPK pathway, structure, disease pathogenesis, and its role as a therapeutic target were covered. As more studies are being performed, we will continue to learn more and understand the AMPK pathway. There remains much to learn about the inner workings of the AMPK pathway, not just as a regulator of the whole body but also as a coordinator of cell growth and metabolism. In view of its protean actions, such work is highly warranted.

### 6.1. AMPK as a Central Metabolic Regulator

AMPK kinase is a master metabolic regulator at the cellular and whole-body organism levels. As noted, AMPK is phosphorylated through stimuli such as glucose, hormones, mitogenic signaling, and energy stress; in response to these stimuli, there is an increase in the energy AMP/ADP levels. Once activated, AMPK induces metabolic changes through the phosphorylation of its substrates. AMPK phosphorylates other pathways for lipid metabolism, mitochondrial biogenesis and autophagy, protein metabolism, and glucose metabolism, all regulators for the body tracing back to the original phosphorylation site of AMPK. An appreciation of AMPK as a central metabolic regulator emerges.

### 6.2. “A Little Goes A Long Way”: Defining the Therapeutic Window of AMPK Activation

The title of this review reflects the emerging concept that the therapeutic benefit of AMPK activation may depend not on achieving maximal or sustained kinase activation, but on restoring an appropriate magnitude, duration, and spatial pattern of AMPK signaling. Physiologically, AMPK is activated dynamically in response to energetic and nutrient stresses, including exercise, fasting, and caloric restriction, thereby promoting ATP-generating pathways while restraining energy-consuming anabolic processes [99,104]. These responses engage downstream pathways involved in fatty acid oxidation, mitochondrial homeostasis, autophagy, and cellular stress adaptation. Accordingly, moderate or temporally restricted AMPK activation may be sufficient to produce metabolic benefits while reducing the potential consequences of prolonged systemic pathway activation [99,104].

Evidence from pharmacological studies supports the existence of a therapeutic window for AMPK modulation, although a universal non-monotonic dose–response relationship has not been established for AMPK activation itself. In mice, long-term administration of a relatively low dose of metformin improved healthspan and lifespan, whereas a substantially higher dose was toxic [101]. Because metformin has multiple AMPK-dependent and AMPK-independent actions, these observations should not be interpreted as direct evidence for a biphasic dose–response relationship of AMPK itself. Nevertheless, they illustrate the broader principle that increasing the intensity of an AMPK-directed metabolic intervention does not necessarily increase therapeutic benefit. Similarly, the potent systemic pan-AMPK activator MK-8722 produced marked improvements in glucose homeostasis in rodents and non-human primates but caused reversible cardiac glycogen accumulation and cardiac hypertrophy during chronic treatment [103]. In contrast, compartment-selective activation of lysosomal AMPK by aldometanib produced metabolic and healthspan benefits without detectable cardiac hypertrophy or dysfunction in treated mice [22]. Collectively, these observations are consistent with a therapeutic-window model in which the consequences of AMPK activation depend not only on activation magnitude and duration but also on tissue distribution, heterotrimer composition, and subcellular localization.

Importantly, the concept that “a little goes a long way” should not be interpreted simply as a recommendation to administer lower doses of all AMPK activators. Drug dose and AMPK target engagement are not equivalent, particularly for indirect activators with multiple molecular targets. Rather, the therapeutic objective should be to achieve the minimum effective and appropriately localized AMPK activation required to engage disease-relevant pathways while avoiding unnecessary activation of AMPK complexes in other tissues [104]. Depending on the indication, this may involve moderate or intermittent activation, partial target engagement, or selective activation of specific tissues, heterotrimeric complexes, or subcellular AMPK pools. Future clinical development should therefore establish pharmacodynamic relationships among drug exposure, AMPK target engagement, downstream pathway activation, therapeutic efficacy, and adverse outcomes rather than assuming that maximal AMPK activation will produce maximal clinical benefit.

This framework provides a rationale for the emerging transition from systemic pan-AMPK activation toward precision AMPK therapeutics. Isoform-, tissue-, and compartment-selective activators may widen the therapeutic window by concentrating AMPK signaling within disease-relevant pathways while minimizing sustained activation in tissues in which excessive or inappropriate AMPK activity may produce undesirable effects [22,103,104]. The cardiac glycogen accumulation and hypertrophy associated with chronic pan-AMPK activation by MK-8722 provide an important example of the potential consequences of indiscriminate pathway activation [103]. Thus, for AMPK-directed therapy, “a little goes a long way” is best understood as a principle of optimized rather than maximal activation. Sufficient AMPK engagement at the appropriate magnitude, location, and duration may provide a more favorable therapeutic index than stronger, prolonged, and indiscriminate pathway activation.

## Figures and Tables

**Table 1:** AMPK subunit isoforms, tissue distribution, and functional characteristics.

Subunit	Gene	Representative Tissue Enrichment/Distribution	Major Functional Features
**α1**	*PRKAA1*	Broadly expressed; prominent in adipose tissue, immune cells, and many non-muscle tissues	Catalytic kinase subunit; Thr172 activation site; frequently associated with cytosolic AMPK signaling
**α2**	*PRKAA2*	Enriched in skeletal muscle, heart, and liver	Catalytic kinase subunit; important for metabolic adaptation during exercise and energetic stress; can exhibit nuclear localization
**β1**	*PRKAB1*	Liver, adipose tissue, pancreas, macrophages, and other tissues	Scaffold subunit; contains carbohydrate-binding module; contributes to ADaM site; targeted by several β1-selective agonists
**β2**	*PRKAB2*	Skeletal muscle and heart	Scaffold and glycogen-binding functions; contributes to ADaM-site pharmacology
**γ1**	*PRKAG1*	Broadly expressed	Major nucleotide-sensing regulatory isoform in many tissues
**γ2**	*PRKAG2*	Prominent in heart; also expressed in other tissues	Mutations cause PRKAG2 cardiomyopathy with glycogen accumulation
**γ3**	*PRKAG3*	Predominantly skeletal muscle	Important in contraction/exercise-associated glucose and glycogen metabolism

**Table 2:** Major upstream regulators of AMPK.

Upstream Regulator	Major Activating Stimulus/Context	Mechanism	AMP/ADP Dependence
**LKB1–STRAD–MO25**	Energetic stress, exercise, mitochondrial dysfunction	Thr172 phosphorylation	Enhanced by AMP/ADP binding
**CaMKK2/CaMKKβ**	Increased intracellular Ca^2+^; neuronal activity; muscle contraction; GPCR signaling	Thr172 phosphorylation	Largely nucleotide-independent
**TAK1–TAB1**	Cellular stress and inflammatory signaling	Context-dependent Thr172 phosphorylation	Context/stimulus dependent
**Lysosomal AXIN–LKB1 complex**	Glucose starvation/reduced FBP	v-ATPase–Ragulator–AXIN/LKB1 assembly at the lysosome	Can occur before detectable whole-cell AMP/ATP changes
**Akt/PKA**	Insulin/growth-factor or cAMP signaling	Ser485/491 inhibitory phosphorylation	Negative regulatory pathway

**Table 3:** Major downstream targets and functions of AMPK.

Pathway	AMPK Target	Major Effect
**Lipid metabolism**	ACC1/ACC2	↓ Fatty acid synthesis; ↑ fatty acid oxidation
**Lipogenic transcription**	SREBP-1c	↓ Lipogenic gene expression
**Cholesterol synthesis**	HMGCR	↓ Cholesterol synthesis
**Cell growth/protein synthesis**	TSC2, RAPTOR	↓ mTORC1 signaling and anabolic growth
**Autophagy**	ULK1	↑ Autophagy and mitophagy
**Mitochondrial biogenesis**	PGC-1α	↑ Mitochondrial biogenesis and oxidative metabolism
**Glucose uptake**	TBC1D1	↑ GLUT4-mediated glucose uptake
**Stress response**	FOXO proteins	↑ Stress-response and antioxidant programs
**Organelle quality control**	TFEB	↑ Lysosomal and mitochondrial quality control

**Table 4:** Representative natural, indirect, and direct AMPK activators.

Compound	Source/Class	Mechanism	AMP/ADP Dependence	Potency	Clinical Status	Key Limitations
**Metformin**	Synthetic biguanide; natural-product-inspired	Complex I inhibition; PEN2–ATP6AP1 lysosomal pathway	Both	Indirect; dose-dependent	Approved for T2DM	AMPK-independent effects
**Berberine**	Coptis/Berberis alkaloid	Complex I inhibition; lysosomal AMPK regulation	Mainly dependent	Indirect; μM cellular range	Human metabolic studies	Low bioavailability; polypharmacology
**Resveratrol**	Grape/berry polyphenol	SIRT1–LKB1; CaMKK2 pathways	Mainly independent	Indirect; μM cellular range	Human studies; supplement	Low bioavailability; multiple targets
**Ginsenosides/Compound K**	Panax ginseng saponins	LKB1/SIRT1-associated AMPK signaling	Mainly indirect/variable	No standardized EC50	Mostly preclinical	Variable absorption and metabolism
**Salicylate**	Plant salicylate; direct activator	β1-AMPK/ADaM-site activation	Independent	Weak; high μM–mM	Approved derivatives for other indications	High concentration; off-target effects
**AICAR**	Synthetic AMP mimetic	Converted to ZMP; binds γ-subunit	AMP-mimetic	~0.1–2 mM in cells	Research agent	Low specificity
**A-769662**	Synthetic direct activator	ADaM-site; β1-selective	Independent	Sub-μM to low-μM	Preclinical	β1 preference; off-target effects
**Compound 991**	Synthetic direct activator	High-affinity ADaM-site activation	Independent	Nanomolar	Preclinical	No clinical development
**Compound 13**	Synthetic direct activator	ADaM-site allosteric activation	Independent	Sub-μM to low-μM	Preclinical	Limited in vivo data
**PF-739**	Synthetic direct pan-activator	Activates multiple AMPK complexes	Independent	Sub-μM to low-μM	Preclinical; rodents/NHPs	Long-term safety unknown
**SC4**	Synthetic direct activator	ADaM-site; complex-selective	Independent	Potent; complex-dependent	Preclinical	Limited in vivo data
**MK-8722**	Synthetic direct pan-activator	Activates all 12 AMPK complexes	Independent	Potent	Preclinical; rodents/NHPs	Cardiac hypertrophy/glycogen
**O304**	Synthetic pharmacological activator	Enhances AMPK; additional mitochondrial effects	Mostly independent	μM cellular range	Phase IIa in T2DM	Mechanism not fully defined

## Data Availability

No new data were created or analyzed in this study. Data sharing is not applicable to this article.
